# Overexpression of the rice gene *OsSIZ1* in Arabidopsis improves drought-, heat-, and salt-tolerance simultaneously

**DOI:** 10.1371/journal.pone.0201716

**Published:** 2018-08-09

**Authors:** Neelam Mishra, Anurag P. Srivastava, Nardana Esmaeili, Wenjun Hu, Guoxin Shen

**Affiliations:** 1 Zhejiang Academy of Agricultural Sciences, Hangzhou, Zhejiang Province, China; 2 Department of Biological Sciences, Texas Tech University, Lubbock, Texas, United States of America; 3 Department of Botany, St. Joseph’s College, Bangalore, India; 4 Department of Chemistry and Biochemistry, Texas Tech University, Lubbock, Texas, United States of America; National Taiwan University, TAIWAN

## Abstract

Sumoylation is one of the post translational modifications, which affects cellular processes in plants through conjugation of small ubiquitin like modifier (SUMO) to target substrate proteins. Response to various abiotic environmental stresses is one of the major cellular functions regulated by SUMO conjugation. SIZ1 is a SUMO E3 ligase, facilitating a vital step in the sumoylation pathway. In this report, it is demonstrated that over-expression of the rice gene *OsSIZ1* in Arabidopsis leads to increased tolerance to multiple abiotic stresses. For example, *OsSIZ1*-overexpressing plants exhibited enhanced tolerance to salt, drought, and heat stresses, and generated greater seed yields under a variety of stress conditions. Furthermore, *OsSIZ1-*overexpressing plants were able to exclude sodium ions more efficiently when grown in saline soils and accumulate higher potassium ions as compared to wild-type plants. Further analysis revealed that *OsSIZ1-*overexpressing plants expressed higher transcript levels of *P5CS*, a gene involved in the biosynthesis of proline, under both salt and drought stress conditions. Therefore, proline here is acting as an osmoprotectant to alleviate damages caused by drought and salt stresses. These results demonstrate that the rice gene *OsSIZ1* has a great potential to be used for improving crop’s tolerance to several abiotic stresses.

## Introduction

Post translational modification of proteins occurs via processes such as methylation, acetylation, phosphorylation, ubiquitination, and sumoylation [1]. Sumoylation is one of the highly conserved post translational modifications in eukaryotes. It is very similar to ubiquitination except that proteins tagged with ubiquitin(s) are likely destined for degradation by the 26S proteasome, whereas SUMO conjugation to target proteins affects various cellular processes in plants including plant’s response to various abiotic stresses [2]. SUMO is attached to target proteins by formation of an isopeptide bond between SUMO’s C terminal Gly residue and Lys residue located in the consensus motif (ψKXE) of target proteins, where ψ is a hydrophobic amino acid residue usually Ile or Val and X can be any amino acid residue [3]. Similar to ubiquitin, SUMOs are synthesized as longer precursors and the glycine at the C-terminus of mature SUMO is exposed by a SUMO-specific isopeptidase. Then, SUMO’s C- terminus is activated by SUMO E1 activating enzyme using ATP, thereafter the activated SUMO is transferred to a cysteine of SUMO E2 conjugating enzyme. Finally, SUMO is ligated to the ε-amino group of a lysine residue in target proteins with the help of SUMO E3 ligase [4, 5].

SIZ/PIAS (SAP and MIZ/Protein Inhibitor of Activated STAT) is first identified and most well characterized SUMO E3 ligase in plants. The SAP, PINIT, SP-RING, SXS motif, and NLS domains are well conserved in all SIZ/PIAS proteins with each domain having a specific function. Additionally, SIZ/PIAS plant homologs possess a plant homeodomain (PHD) that is a zinc finger [6]. Three additional E3 ligases have been identified in plants: RanBP2 (Ran binding protein), Pc2 (polycomb group), and NSE2/MMS21 (non-SMC element/methyl methanesulfonate sensitive). Very recently MMS21 from *Arabidopsis thaliana* has been characterized and is known to regulate drought tolerance in *Arabidopsis thaliana* [7].

Mutations in genes that encode E1 activating or E2 conjugating enzymes cause embryonic lethal phenotypes, suggesting that these genes are important for plant viability [8]. SUMO proteases, enzymes responsible to recycle free SUMO, are known to be involved in several regulatory pathways in plant development [9–12], plant pathogen interaction for altering host cell defense [13], as well as in plant salt tolerance [14, 15]. Furthermore, it has been found that the SIZ1 is known to regulate various vital processes in plants, one of which is response to several abiotic stresses. Most of studies reported till date have utilized knockout mutants to study the function of gene SIZ1. There is only one SIZ1 gene in Arabidopsis (i.e. *AtSIZ1*), and knockout mutants were found to be dwarf with increased *PR1* (Pathogenesis Related gene 1) expression, resistance to bacterial pathogens with an increased level of salicylic acid and hypersensitivity to phosphorus limited conditions [16].

Rice homologs of AtSIZ1, OsSIZ1 and OsSIZ2, share 51% and 43% sequence identity with AtSIZ1 at the amino acid level, respectively [17]. Additionally, [17] showed that *OsSIZ1* and *OsSIZ2* partially complement the ABA hypersensitive phenotype of the At*siz1* mutant. *AtSIZ1* directly regulates plant growth and development through regulation of salicylic acid signaling [11], progression of cell cycle [18], and cytokinin signaling affecting proliferation of root cells [19, 20]. *AtSIZ1* regulates salicylic acid signaling which in turn affects several processes such as cell division, expansion, flowering and innate immunity [21–23].

Besides these, AtSIZ1 negatively regulates abscisic acid (ABA) signaling via sumoylation of ABI5 [24], modulates the expression of various ABA and other abiotic stress responsive genes [25, 26]. siz1 knockout mutants showed enhanced ABA sensitivity and ABA induced gene expression. With the help of knockout mutant study it was found that SIZ1 is involved in sumoylation of nitrate which in turn positively affects nitrogen assimilation [27]. Many studies have shown that SIZ1 is involved in tolerance to various environmental stresses with the use of reverse genetics. Most of the studies were conducted on Arabidopsis SIZ1. AtSIZ1 is also known to regulate plant response to various environmental stresses including drought tolerance [28], heat tolerance [29, 30] low temperature tolerance via sumoylation of ICE1 [31], phosphate (Pi) starvation via sumoylation of PHR1 (a myb transcription factor) [32], excess copper tolerance [33, 34], and salt stress tolerance [35, 36] reported that overexpression of *OsSIZ1* in creeping bentgrass enhances resistance to several abiotic stresses such as heat, drought, and Pi starvation. Recently, it has been demonstrated that overexpression of *OsSIZ1* in cotton improves both drought and heat tolerance [37].

With all the studies doe till date, it is evident that SIZ1 is potentially involved in enhancing plant’s tolerance to several environmental stresses. Under natural conditions, plant survival is often challenged by two or more stresses at the same time [38, 39]. Multiple stress tolerance can be achieved by using different strategies. One approach is the gene pyramiding approach [40], where co-expression of two or more genes concurrently can further increase multi-stress tolerance, second approach is to express transcription factor genes such as *NAC*s [41] or genes that can provide tolerance towards multiple stresses such as the *mtID* gene [42]. In the present study, we showed that overexpression of *OsSIZ1* in Arabidopsis improves tolerance to drought, heat, and salt stress as transgenic plants were able to maintain higher chlorophyll content and generated greater seed yield than wild-type plants under these stresses individually or in combinations. Additionally, we have observed that, when grown under saline conditions, *OsSIZ1*-transgenic plants accumulate less sodium ions in cytoplasm than wild-type plants, thereby avoiding the toxic effects of sodium ions in cytoplasm. Moreover, it was also observed that under both drought and salt stress conditions, *O*s*SIZ1-*transgenic plants expressed higher transcript level for the pyrroline 5-carboxylate synthase (a rate limiting enzyme in proline biosynthesis) than wild-type plants. This is an indication that enhanced performance of transgenic plants under salt and drought stresses might be due to synthesis of higher amounts of osmo-protectant, i.e. proline, which is consequently responsible for maintaining a low water potential in plant cells. Therefore, we believe that *SIZ1* can be used to improve multiple-stress tolerance in crop plants.

## Materials and methods

### Plant transformation

The Ubi-OsSIZ1 construct [36] provided to us by Dr. Hong Luo, Clemson University was transformed into *Agrobacterium* strain GV3101 and then the transformed strain containing the *OsSIZ1* gene was used to transform wild-type (WT) Arabidopsis (Col-0) using the “floral dip” method [43]. The cauliflower mosaic virus 35S promoter driven *bar* gene coding for herbicide glufosinate ammonium (BASTA) resistance was used for selection of transgenic plants. A total of 20 independent transgenic lines were chosen for further analysis and homozygous plants were obtained by selecting the T_2_ seeds on Murashige and Skoog (MS) media [44] supplemented with 30 μg ml^-1^ of BASTA (for selection of transgenic plants).

### Plant growth conditions

Arabidopsis seeds (>100) were surface sterilized with 15% bleach for 20 min followed by 3–5 times washes in sterilized distilled water. After four days of stratification at 4 ^o^C in darkness, seeds were plated onto the MS media supplemented with 30 μg ml^-1^ BASTA and then herbicide resistant plants were transferred from plates to soil and kept in growth chamber with the 16 h/8 h (light/night) photoperiod. Homozygous T_3_ and T_4_ plants were used for all stress physiology experiments.

### RNA blot and DNA blot analyses

Ten day-old Arabidopsis plants were ground in liquid nitrogen, and total RNAs were extracted by using the TRIzol reagent (Invitrogen). Ten micrograms of the extracted RNAs were run on 1.2% (w/v) denaturing agarose gel and then transferred onto a BioTrans ^(+)^ nylon membrane (ICN Biochemicals). After cross-linking, nylon membrane was hybridized with P^32^ labelled probes (*OsSIZ1* and *Actin 8*) under the condition described by [45]. After hybridization, nylon membranes were washed and then exposed to a Phosphor Imager screen for 24 h before data were collected. The *Actin 8* gene was used as the RNA loading control.

Genomic DNA was extracted using the CTAB DNA extraction method with minor modifications [46]. Ten micrograms of DNA from both WT and two *OsSIZ1-*overexpressing lines were digested with *Eco* RI in 400 μl reaction volume according to the protocol (NEB, MA, and USA). After overnight digestion, DNA was precipitated using ethanol and dissolved into 33 μl of water, and mixed with 7 μl of 6X loading dye. The resultant 40 μl solution were loaded into 0.8% agarose gel for electrophoresis overnight. Thereafter, membrane transfer and DNA hybridization were performed as previously described [47]. Two cDNA fragments of *OsSIZ1* were used as the probes for DNA hybridization.

### Preparation of radioactive labels and hybridization conditions

Gene-specific probes used for RNA blot analyses were designed as SIZ1-1, SIZ1-2 and Actin 8 that have lengths of 1056 bp, 663 bp, and 502 bp, respectively. The pHL080*SIZ1* construct was used as the template to amplify the two *OsSIZ1* fragments (i.e. SIZ-1 and SIZ-2) via PCR. An Arabidopsis cDNA library was used to amplify the *Actin 8* gene. Probe for DNA blot analysis was made by using the *OsSIZ1* gene fragments amplified by PCR. Two PCR products of *OsSIZ1* were gel-purified and mixed in equal amounts and then used as templates for making probes. Random priming reaction was performed with the DECAprime™ II DNA Labeling Kit (Life Technologies) to radio-chemically label the PCR fragments using ^32^P-α-dATP as the ^32^P donor. The oligonucleotide primers used were ACT8_F and ACT8_R for *Actin8*, SIZ1-1_F, SIZ1-1_R and SIZ1-2_F, SIZ1-2_R for *OsSIZ1*. The hybridization conditions for both RNA blot analysis and DNA blot analysis were essentially the same as those described by [45].

### Effects of heat stress on plant survival and reproductive development

To examine the effects of heat stress on hypocotyl elongation in darkness, stratified seeds were plated vertically on MS media and kept in darkness for 3 days and then plates were transferred to a temperature controlled incubator set at 45 ^o^C for 5 h. After heat shock, plates were transferred back to a temperature of 22 ^o^C in darkness and hypocotyl length was recorded one week after recovery under normal conditions (22 ^o^C). To study the basal heat tolerance response in early seedling stages, seedling survival assay was done as described by [48]. Five days old horizontally grown seedlings on MS media were heat shocked (45 ^o^C for 20 min) in a temperature controlled water bath and seedling survival was scored after a 7-day recovery period under normal conditions, and represented as a percentage of viable seedlings. Seedlings that were still green and producing new leaves were scored as surviving.

For heat stress treatment in soil, 4 weeks after germination, pots containing seedlings were transferred to a growth chamber that was set at 16 h/8 h photoperiod, 22 ^o^C for 19 h and 37 ^o^C for 5 h every day (12:00 noon– 5:00 pm). Pictures were taken 2 months after heat stress was started and rosette leaf samples were harvested for biochemical analyses. At the end of experiment, seed yield was measured for both WT and *OsSIZ1-*transgenic lines. Seed collectors were used to ensure no seed loss before harvesting. Experiment was repeated three times with 6 biological replicates for each genotype.

### Effect of salinity stress on root growth and seed yield

For salt stress treatment, seeds were sterilized and stratified as described above and plated vertically on MS media. After 3 days, seedlings were transferred to MS media supplemented with 125 mM and 150 mM NaCl, respectively. Root lengths were measured 12 days after transferring to plates containing 125 mM NaCl. However, for plates supplemented with 150 mM NaCl, fresh weight of ten seedlings were measured one week after transferring for both WT and two *OsSIZ1-*transgenic lines as no significant root growth could be seen after transfer. For salt stress treatments in soil, three and a half weeks after germination, salt was applied incrementally from 50 mM NaCl (100 ml per pot, twice in 6 days), to 100 mM (twice in the next 6 days), and to 150 NaCl (three times in the next 9 days). Phenotypic differences were documented 21 days after incremental salt stress treatment and rosette leaves were sampled for biochemical analysis and RNA extraction for real time PCR experiments. Thereafter, plants were watered for 4 weeks with regular water and seed weight were measured for all genotypes at the end of experiment. Additionally, Na^+^ and K^+^ ion contents in roots and shoots were analyzed for plants grown under both control and salt stress conditions as described by [49]. All experiments were repeated three times with 6 biological replicates for each genotype.

### Effects of drought stress on seed germination and seed yield

To examine the effect of drought stress on seed germination, seeds were plated on MS media containing 0.05 M (40%) polyethylene glycol (PEG-8000) and germination was scored every day for 2 weeks. PEG reduces water potential in plant growth media, creating a dehydration condition (osmotic stress) for plant growth. PEG containing plates were prepared according to the protocol from [50]. For long term assays, WT and two *OsSIZ1*-transgenic lines were grown under 22 ^o^C, 16 h/8 h photoperiod in growth chamber for 3.5 weeks, and then watering was stopped for two weeks and rosette leaves were sampled for biochemical analysis and RNA extraction for real time PCR experiments. Pots were weighed before starting the drought stress to ensure that same amount of water is present in all pots. After two weeks of drought stress (no watering) treatment, plants were re-watered, then one day later plants were watered every two days, as opposed to control plants that were watered every other day until harvesting. At the end of experiment, seed yield was measured for all plants. Experiments were repeated three times with five biological replicates.

### Effects of combined heat and salt stress on root growth and seed yield

For combined heat and salt stress treatment, seeds were directly plated on the MS media supplemented with 100 mM NaCl and kept in growth chamber that was set at 30 ^o^C and 16 h/8 h photoperiod. After 18 days, pictures were taken to document the phenotypic differences between WT and the two *OsSIZ1*-transgenic lines, and root length was measured for all genotypes. Additionally, plants were sown in soil to determine the long-term effect of combined stresses of heat and salt. Four and a half weeks after germination, plants were transferred to growth chamber that was set at 22 ^o^C for 19 h and 37 ^o^C for 5 h (12:00 noon -5:00 pm) per day with a photoperiod of 16 h light and 8 h night. Salt was applied incrementally starting from 50 mM NaCl (150 ml per pot, three times in 6 days), to 100 mM (three times in the next 6 days), and thereafter plants were watered with normal water until harvesting. Pictures were taken 18 days after the start of incremental salt stress treatments to document the phenotypic differences between the WT and *OsSIZ1-*transgenic plants. Survival rate and silique number were recorded at the end of experiment. All experiments were repeated three times with 6 biological replicates.

### Effects of combined stresses of heat and water deficit on germination and seed yield

To study the effect of combined stresses of heat and water deficit on germination, sterilized and stratified seeds were plated on the MS media supplemented with 300 mM of mannitol and kept for 48 h in an incubator that was set at 37 ^o^C. After 48 h, plates were transferred to normal condition (22 ^o^C). Germination was scored every day for 14 days. To study the effect of combined stresses of heat and water deficit on plants in soil, seeds were sown in soil and grown for 4.5 weeks, then plants were transferred to a growth chamber that was set at 22 ^o^C for 19 h and 37 ^o^C for 5 h (12:00 noon -5:00 pm) per day with a photoperiod of 16 h light and 8 h night. Then watering was stopped for one week and pictures were taken to document the phenotypic differences. After one week of no irrigation, plants were re-watered and recovered for one day. Subsequently, the stress treated plants were watered every two days as opposed to control plants that were watered every other day until harvesting. Survival rate and silique number were recorded at the end of experiment. Experiment was repeated twice with 7 biological replicates.

### Effects of non-ionic osmotic stress on root length

To study the effect of non-ionic osmotic stress on root growth, seedlings vertically grown on MS media for 3 days were transferred to MS media supplemented with or without 300 mM mannitol, and then root lengths were measured 12 days after transferring and pictures were taken. Experiment was repeated three times with 8 seedlings for every genotype.

### Analyses of biochemical compounds under control, heat, salt, and drought stress conditions

Chlorophyll content was measured for control and stressed (salt, drought, and heat) leaves using the methanol extraction method [51]. Samples with a hundred mg of leaf materials were incubated in cold methanol for 24 h at 4°C. The supernatant solution was poured off into a new container, and 1 ml of this sample was analyzed spectrophotometrically at 652 nm and 665.2 nm to measure the concentration of chlorophyll *a* and *b*. The amount of chlorophyll *a* and *b* were calculated using the following formula based on the molar extinction coefficient of these molecules.

$$\mathrm{Amount}\;\mathrm{of}\;\mathrm{chlorophyll}\;a\;\left({\mu g\;\mathrm{ml}}^{‑1}\right)=16.29A_{665.2}‑8.54A_{652.0}$$

$$\mathrm{Amount}\;\mathrm{of}\;\mathrm{chlorophyll}\;b\;\left({\mu g\;\mathrm{ml}}^{‑1}\right)=30.66A_{652}‑13.58A_{665.2}$$

Anthocyanin content was determined as described by [52]. Briefly, around 200 mg of leaf samples were ground in the methanol-HCl solution followed by dilution using two thirds volume of distilled water. To the aqueous phase, equal volume of chloroform was added. The solution was then thoroughly mixed and centrifuged at 10,000 g for 1 min. Absorbance of aqueous phase containing anthocyanin was measured at 535 nm.

Lipid peroxidation was estimated in terms of malondialdehyde (MDA) content as described by [53]. Samples with a hundred mg of leaf materials were homogenized in 0.1% TCA followed by centrifugation at 15,000 g for 10 min at 4 ^o^C. Four volumes of 0.5% TBA in 20% TCA was added to one volume of supernatant and the mixture was incubated at 95 ^o^C for 25 min. The reaction was terminated by incubating the mixture on ice for 15 min and then absorbance was measured spectrophotometrically at 532nm and 600nm. The amount of MDA was calculated using an extinction coefficient of 155 mM^-1^cm^-1^. All biochemical experiments were conducted with two biological and three technical replicates.

Proline content was measured as described by [54]. Fresh rosette leaves (0.5 g) were homogenized in 10 ml of 3% sulphosalicylic acid and then centrifuged at 10,000*g*. The supernatant (0.5 ml) was mixed with 1 ml of glacial acetic acid and 1 ml of 2.5% acid ninhydrin (2.5 g of ninhydrin dissolved in a mixture of 60 ml glacial acetic acid and 40 ml 6 M phosphoric acid). The mixture was incubated for 1 h at 100°C and then the reaction was terminated by cooling in an ice bath. The reaction mixture was extracted with 2 ml of toluene, mixed vigorously with the test tubes stirrer for 15 s. The chromophore-containing toluene was warmed to room temperature and absorbance was measured at 520 nm using toluene as a blank. The proline concentration was determined from standard curve and calculated per g of fresh weight.

### Real time (quantitative) PCR analysis of the transcripts of abiotic stress responsive genes

RNAs were extracted from leaves of soil grown Arabidopsis plants (control and stressed) and then treated with RQ1 DNase (Promega) at 37 ^o^C for 30 min to remove potential DNA contamination. Then RNA was isolated using phenol chloroform extraction and precipitated overnight at -20 ^o^C using equal volume of ice-cold isopropanol. The RNA pellet was washed twice with 70% ethanol and then dissolved into DEPC-treated water. Two micrograms of RNAs were used for first strand cDNA synthesis using Super Script® VILO™ Master Mix. The PCR reaction consisted of 25 ^o^C for 10 min, 42 ^o^C for 1 h followed by termination of reaction at 85 ^o^C for 5 min. Real-time PCR was conducted with the PCR machine 7500 sequence detection system (Applied Biosystems), using Thermo Scientific Absolute qPCR SYBR Green Fluorescein Mix. PCR was performed with an initial denaturation at 95 ^o^C for 3 min followed by 40 cycles of denaturation at 95 ^o^C for 15 s and extension at 55 ^o^C for 40 s. Experiment was conducted with two biological and three technical replicates. The oligonucleotide primers used were Actin8_qF and Actin8_qR for *Actin8*, RD29A_F and RD29A_R for *RD29A* (At5G52310), RD29B_F and RD29B_R for *RD29B* (At5G52300), NCED3_F and NCED3_R for *NCED3* (At3G14440), and P5CS_F and P5CS_R for *P5CS* (At3g55610) genes.

### Statistical analysis

Student *t*-test considering one tailed unequal variance was performed to compare the performance of WT and *OsSIZ1*-transgenic lines. All *P* values were from comparison between controls (WT) and *OsSIZ1*-transgenic plants. Statistical analysis was performed using Microsoft® Office Excel 2007. *P < 0.05 and **P < 0.01, are the two significance level shown in the data presented in Figs 1–10.

**Fig 1:**
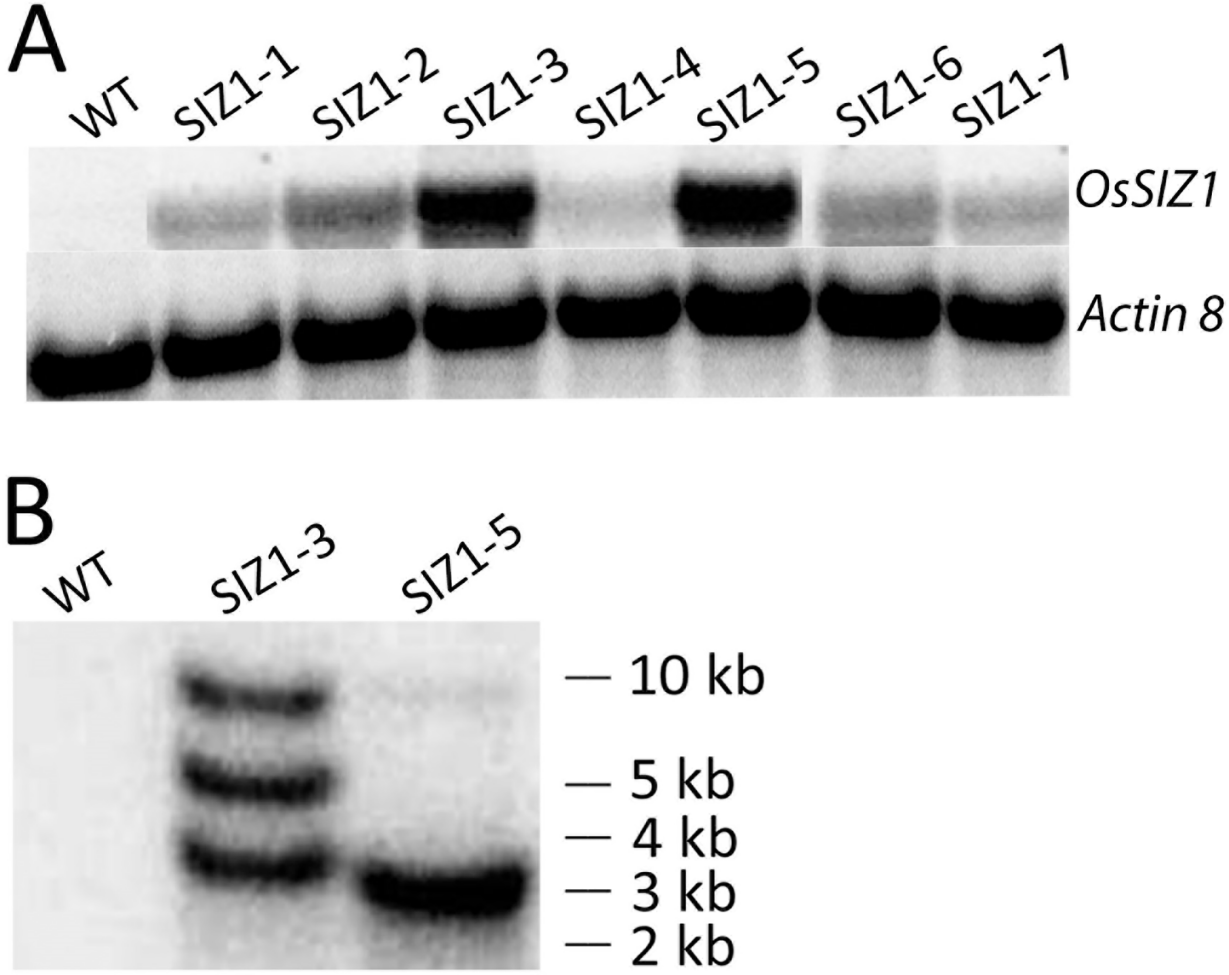
Molecular analysis of *OsSIZ1*-transgenic plants. **A.** RNA blot analysis of wild-type and seven independent *OsSIZ1-*transgenic lines. **B.** DNA blot analysis of wild-type and two high expression lines of *OsSIZ1*-transgenic plants. WT, wild-type; SIZ1-3 and SIZ1-5, two independent *OsSIZ1*-transgenic plants.

**Fig 2:**
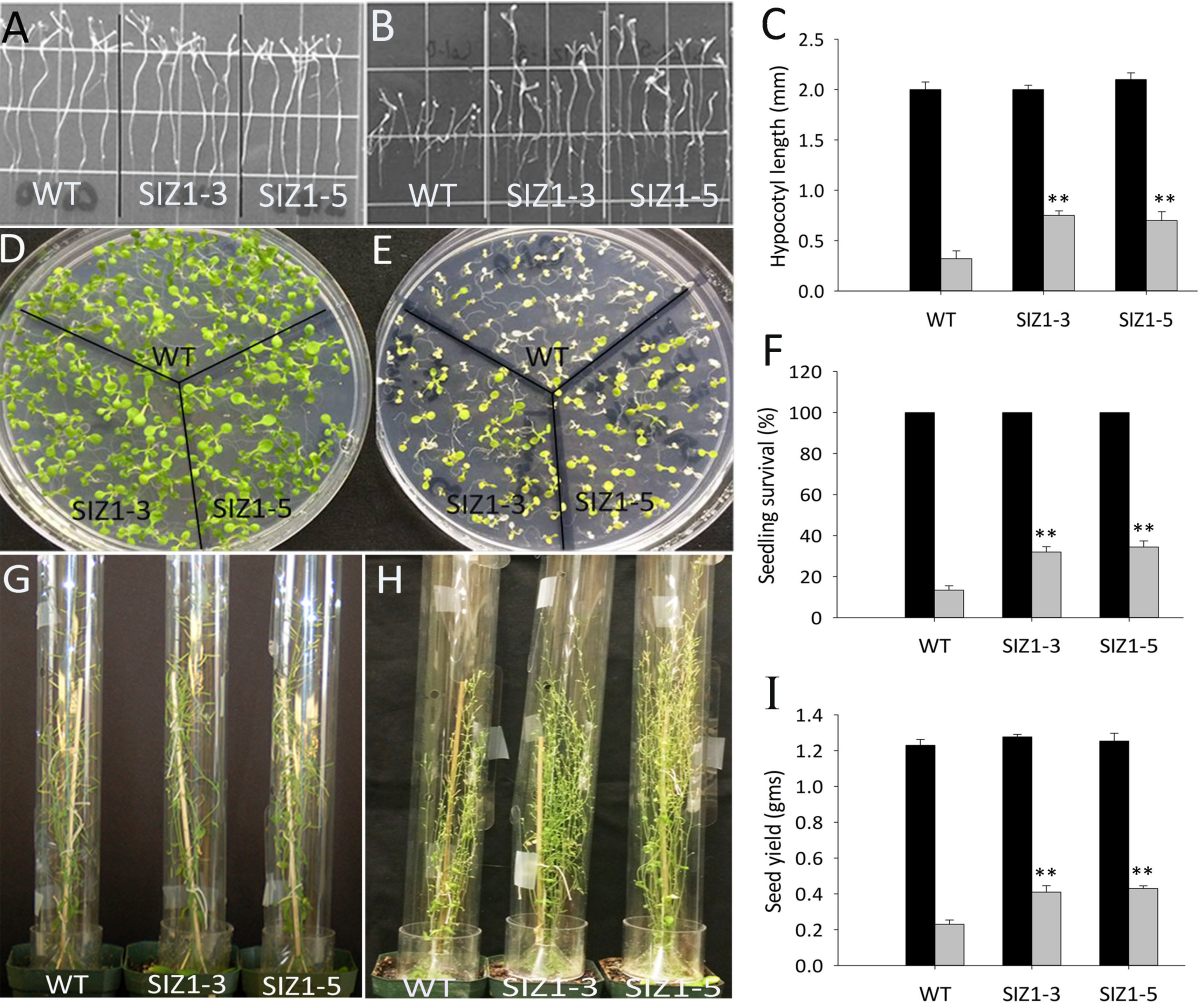
Phenotypes of wild-type and *OsSIZ1*-transgenic plants under normal temperature and after heat stress treatment. **A.** Phenotypes of wild-type and two independent *OsSIZ1*-transgenic plants under normal growth condition (i.e. 22 ^o^C). Plants were grown vertically on MS media in darkness for 9 days before the picture was taken. **B.** Phenotypes of wild-type and two independent *OsSIZ1*-transgenic plants after heat treatment. Plants were grown vertically on MS media in darkness for 3 days and then exposed to heat shock at 45 ^o^C for 5 h, then returned to normal growth condition for 6 days before the picture was taken. **C.** Analysis of hypocotyl length of wild-type and two independent *OsSIZ1* transgenic lines shown in figure A and B. Black column, normal condition; grey column, after heat shock treatment. **D.** Phenotypes of wild-type and two independent *OsSIZ1-*transgenic plants under normal growth conditions. Plants were grown on MS media for 12 days before the picture was taken. **E.** Phenotypes of wild-type and two independent *OsSIZ1-*transgenic plants under after heat treatment. Plants were grown on MS media for 5 days and then exposed to 45 ^o^C for 20 min, then plants were returned to normal growth condition for 7 days before the picture was taken. **F.** Survival rates of plants shown in figure D and E. Black column, under normal condition; grey column, after heat shock treatment. **G.** Phenotypes of wild-type and two independent *OsSIZ1-*transgenic plants grown under normal growth condition. Picture was taken 10 weeks after germination. **H.** Phenotypes of wild-type and two independent *OsSIZ1-*transgenic plants after heat stress treatment. Plants were exposed to heat stress (37 ^o^C for 5 h every day) 4 weeks after germination and the picture was taken 6 weeks after the heat stress was started. **I.** Seed yields of wild-type and two independent *OsSIZ1-*transgenic plants grown under normal growth condition or after heat stress treatment. Black column, normal condition; grey column, after heat stress treatment. ** statistically significant at 1%; WT, wild-type; SIZ1-3 and SIZ1-5, two independent *OsSIZ1*-transgenic lines.

**Fig 3:**
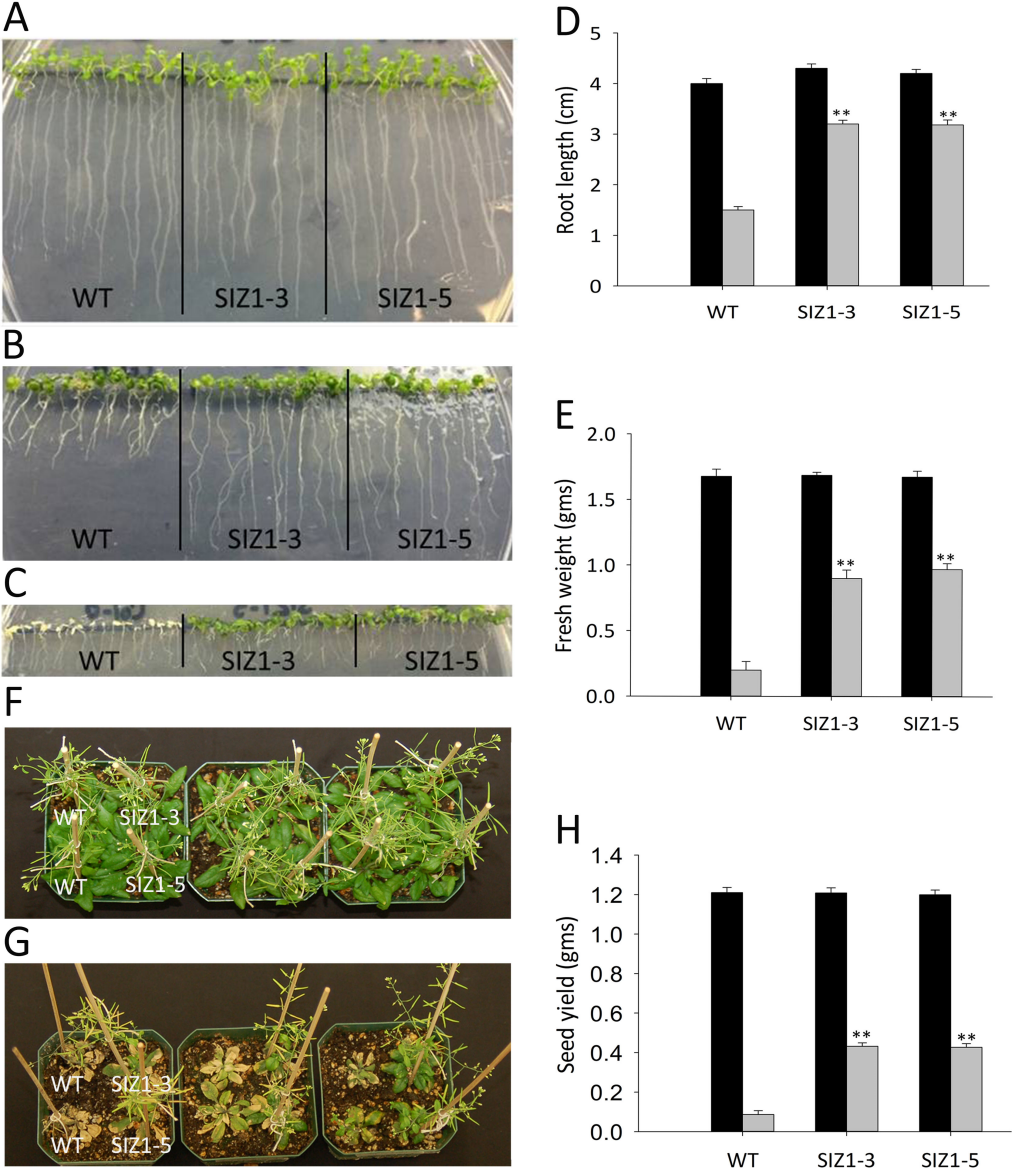
Phenotypes of wild-type and *OsSIZ1-*transgenic plants under normal growth condition and after salt stress treatment. **A.** Phenotypes of wild-type and two independent *OsSIZ1*-transgenic plants on MS media. Plants were grown vertically for 8 days before the picture was taken. **B.** Phenotypes of wild-type and two independent *OsSIZ1*-transgenic plants on salt-containing MS media. Three days old seedlings on MS media were transferred to MS media containing 125 mM NaCl and were allowed to grow vertically for 5 days before the picture was taken. **C.** Phenotypes of wild-type and two independent *OsSIZ1*-transgenic plants on salt-containing MS media. Three days old seedlings on MS media were transferred to MS media containing 150 mM NaCl and were allowed to grow vertically for 5 days before the picture was taken. **D.** Analysis of root length of wild-type and two independent *OsSIZ1*-transgenic plants on MS media or after salt treatment for 5 days. Black column, MS media; grey column, MS media supplemented with 125 mM NaCl. **E.** Analysis of fresh weight of wild-type and two independent *OsSIZ1*-transgenic plants on MS media or after salt treatment for 7 days. Ten seedlings of each genotype were used in the analysis. Black column, MS media; grey column, MS media supplemented with 150 mM NaCl. **F.** Phenotypes of wild-type and two independent *OsSIZ1-*transgenic plants grown under normal growth condition for 42 days. **G.** Phenotypes of wild-type and two independent *OsSIZ1*-transgenic plants 21 days after salt stress was started. **H.** Seed yields of wild-type and two independent *OsSIZ1*-transgenic plants grown under normal growth condition or after salt stress treatment. Black column, normal condition (irrigation with regular water); grey column, salt stress condition (irrigation with incremental salt concentrations in water). ** statistically significant at 1%; WT, wild-type; SIZ1-3 and SIZ1-5, two independent *OsSIZ1*-transgenic plants.

**Fig 4:**
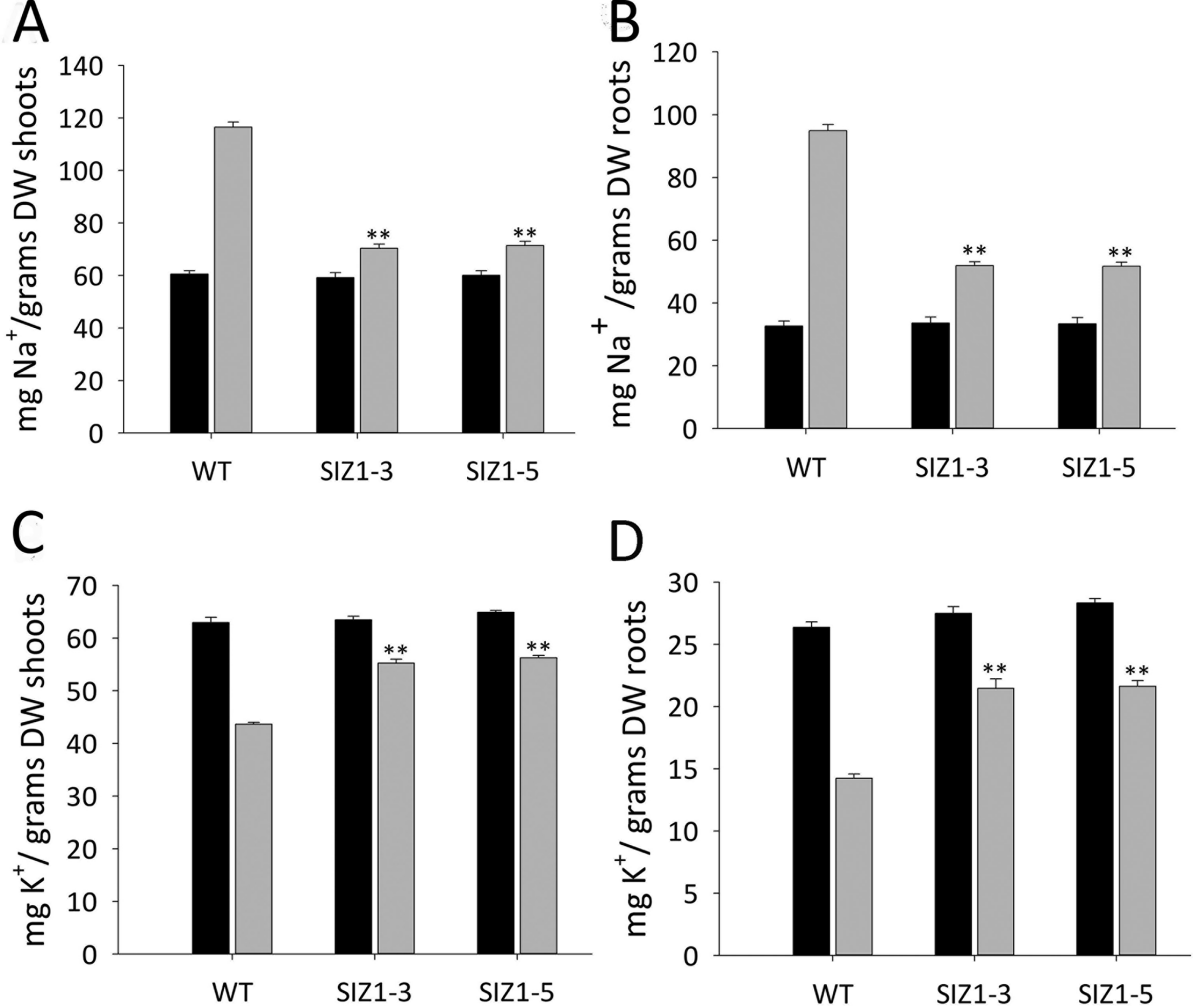
Sodium and potassium ion contents in the roots and shoots of wild-type and *OsSIZ1*-transgenic plants grown under normal and salt stress conditions. **A.** Na^+^ content in shoots of wild-type and two independent *OsSIZ1*-transgenic plants grown under normal and salt stress conditions. **B.** Na^+^ content in roots of wild-type and two independent *OsSIZ1*-transgenic plants grown under normal and salt stress conditions. **C.** K^+^ content in shoots of wild-type and two independent *OsSIZ1*-transgenic plants grown under normal and salt stress conditions. **D.** K^+^ content in roots of wild-type and two independent *OsSIZ1*-transgenic plants grown under normal and salt stress conditions. Black column, normal condition (irrigation with regular water); grey column, salt stress condition (irrigation with incremental salt concentrations in water). ** statistically significant at 1%; WT, wild-type; SIZ1-3 and SIZ1-5, two independent *OsSIZ1* transgenic plants.

**Fig 5:**
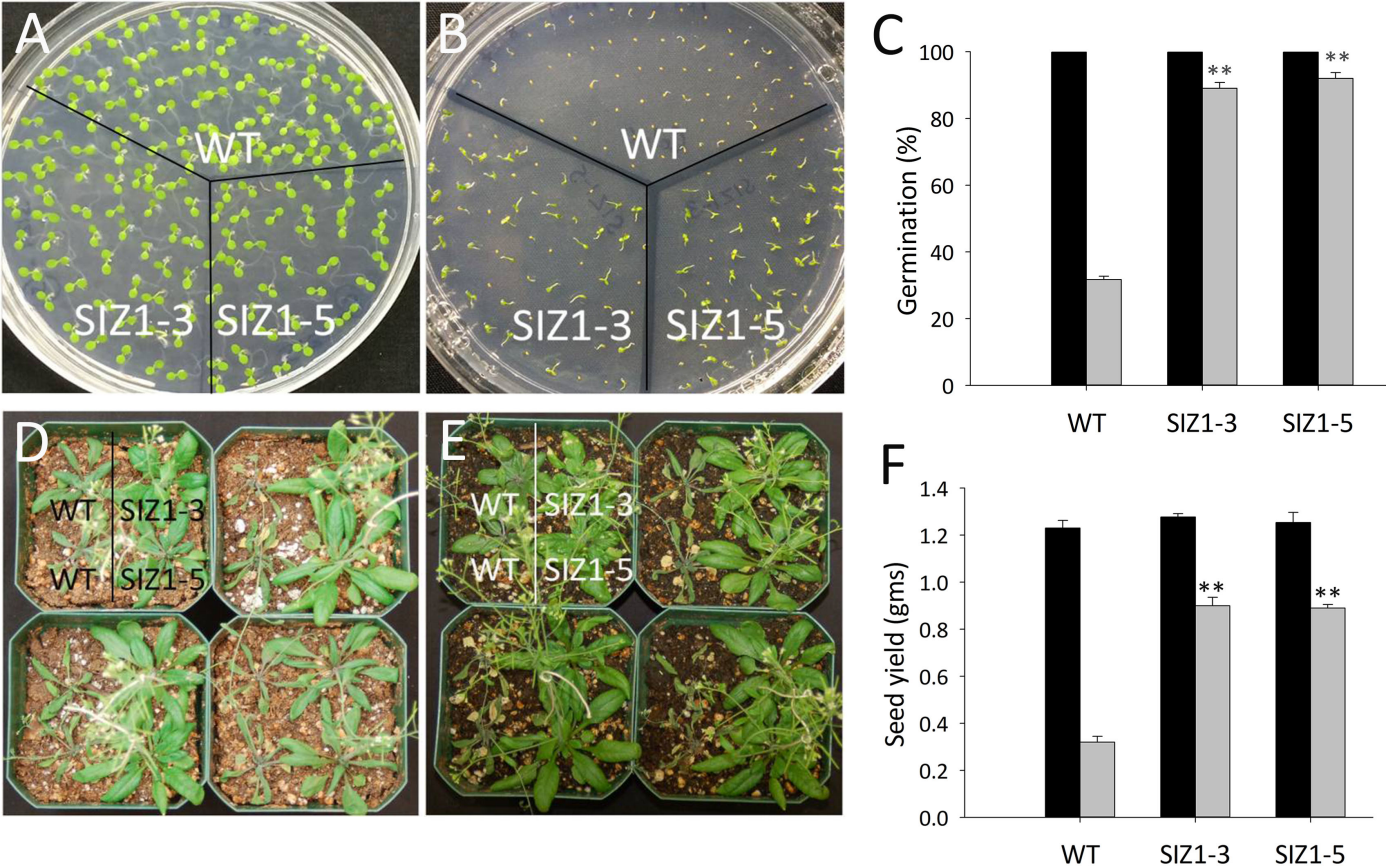
Phenotypes of wild-type and two independent *OsSIZ1*-transgenic plants under normal growth and water deficit stress conditions. **A.** Phenotypes of wild-type and two independent *OsSIZ1*-transgenic plants on MS media. Plants were grown on MS media for two weeks before the picture was taken. **B.** Phenotypes of wild-type and two independent *OsSIZ1*-transgenic plants on media containing PEG-8000. Plants were grown on MS media containing PEG-8000 for two weeks before the picture was taken. **C.** Germination of wild-type and two independent *OsSIZ1*-transgenic plants on MS media or MS media containing PEG-8000. Plants were allowed to grow for 2 weeks before germination was scored. Black column, normal condition (MS media); grey column, water deficit condition (MS media containing PEG-8000). **D.** Phenotypes of wild-type and two independent *OsSIZ1*-transgenic plants after water deficit treatment. Plants were withheld water for 2 weeks. **E.** Phenotypes of wild-type and two independent *OsSIZ1*-transgenic plants one day after re-irrigation following water deficit treatment. **F.** Seed yields of wild-type and two independent *OsSIZ1*-transgenic plants under normal condition (with regular irrigation) and after water deficit treatment (no watering for two weeks). Black column, normal condition (full irrigation); grey column, water deficit condition (no watering for 2 weeks). ** statistically significant at 1%; WT, wild-type; SIZ1-3 and SIZ1-5, two independent *OsSIZ1*-transgenic plants.

**Fig 6:**
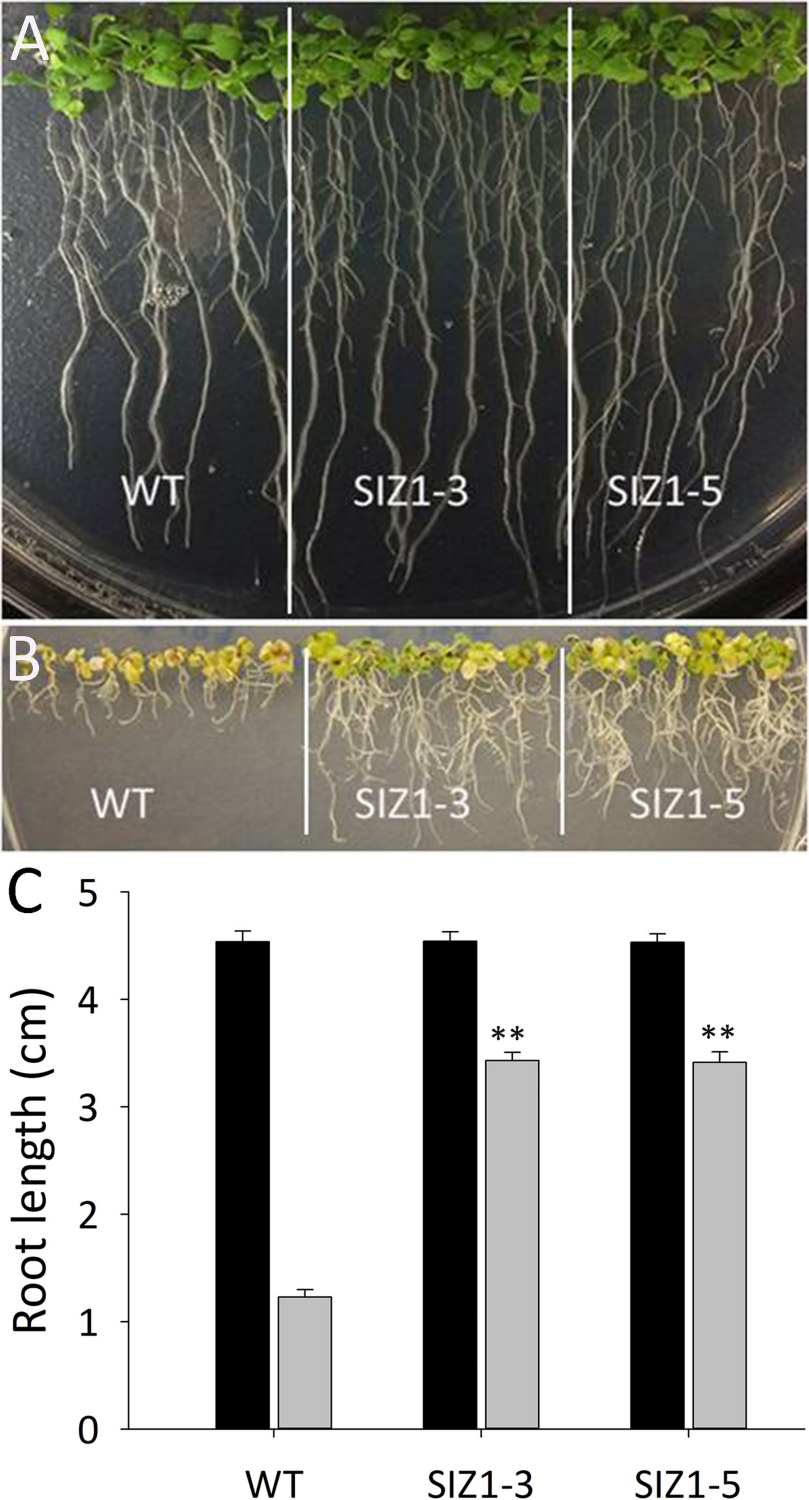
Performance of wild-type and two independent *OsSIZ1-*transgenic plants under combined stresses of heat and salt on MS media. **A.** Phenotypes of wild-type and two independent *OsSIZ1*-transgenic plants under normal growth condition. Plants were grown on MS media vertically for 12 days at 22 ^o^C. **B.** Phenotypes of wild-type and two independent *OsSIZ1*-transgenic plants under combined stresses of heat and salt. Plants were grown on salt-containing MS media (100 mM NaCl) at 30 ^o^C for 18 days. **C.** Analysis of root lengths of wild-type and *OsSIZ1*-transgenic plants under normal growth condition and combined stresses of heat and salt. Plants were grown on MS media at 22 ^o^C for 12 days and then grown on MS media with 100 mM NaCl at 30 ^o^C for 18 days. Black columns, root lengths under normal condition; grey columns, root lengths under combined stresses of heat and salt. ** statistically significant at 1%; WT, wild-type; SIZ1-3 and SIZ1-5, two independent *OsSIZ1*-transgenic plants.

**Fig 7:**
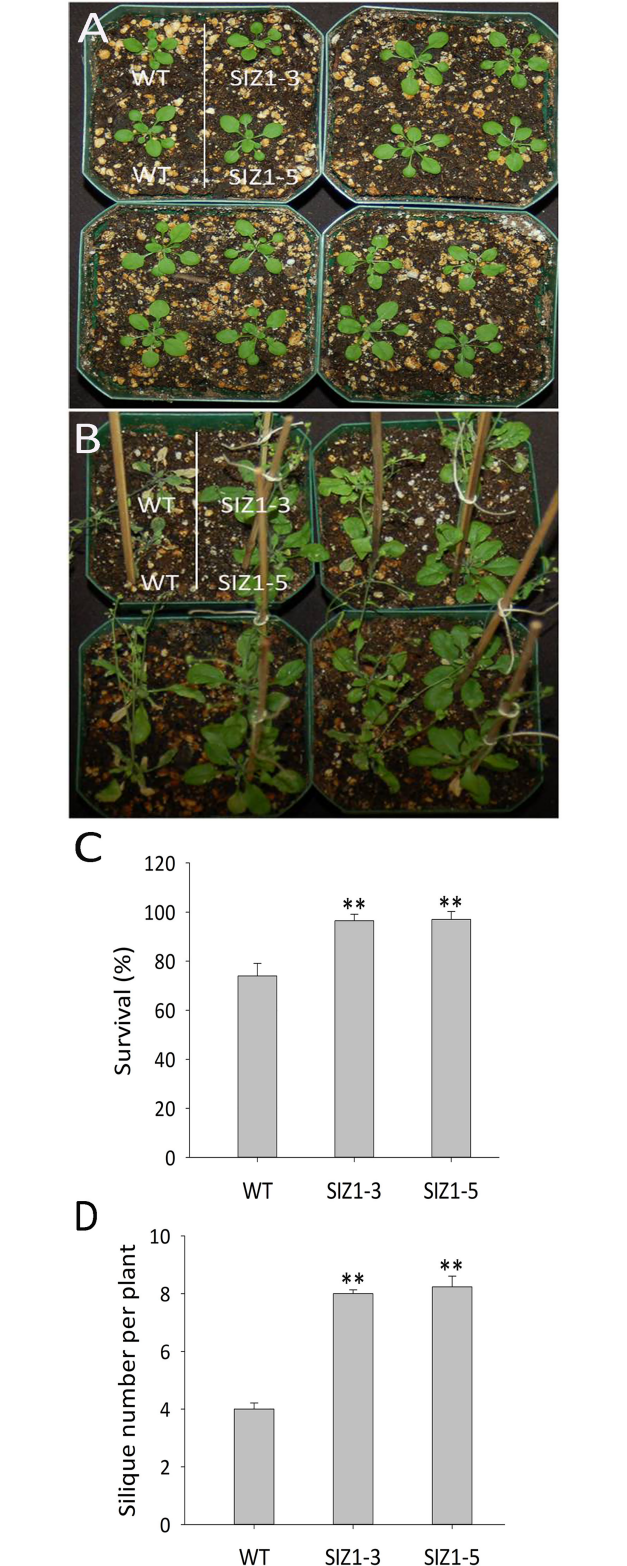
Performance of wild-type and two independent *OsSIZ1-*transgenic plants under combined stresses of heat and salt in soil. **A.** Phenotypes of wild-type and two independent *OsSIZ1*-transgenic plants before stresses were started. **B.** Phenotypes of wild-type and two independent *OsSIZ1*-transgenic plants under combined stresses of heat and salt. On day 28 after germination, plants were exposed to combined stresses of heat and salt (50 mM NaCl three times in 6 days, 100 mM NaCl three times in the next 9 days, and temperature of 37 ^o^C for 5 h every day), and the picture was taken 18 days after the start of combined stresses. **C.** Survival rates of wild-type and two independent *OsSIZ1*-transgenic plants after the treatment of combined stresses of heat and salt. **D.** Silique numbers of wild-type and two independent *OsSIZ1*-transgenic plants after the treatment of combined stresses of heat and salt. ** statistically significant at 1%; WT, wild-type; SIZ1-3 and SIZ1-5, two independent *OsSIZ1*-transgenic plants.

**Fig 8:**
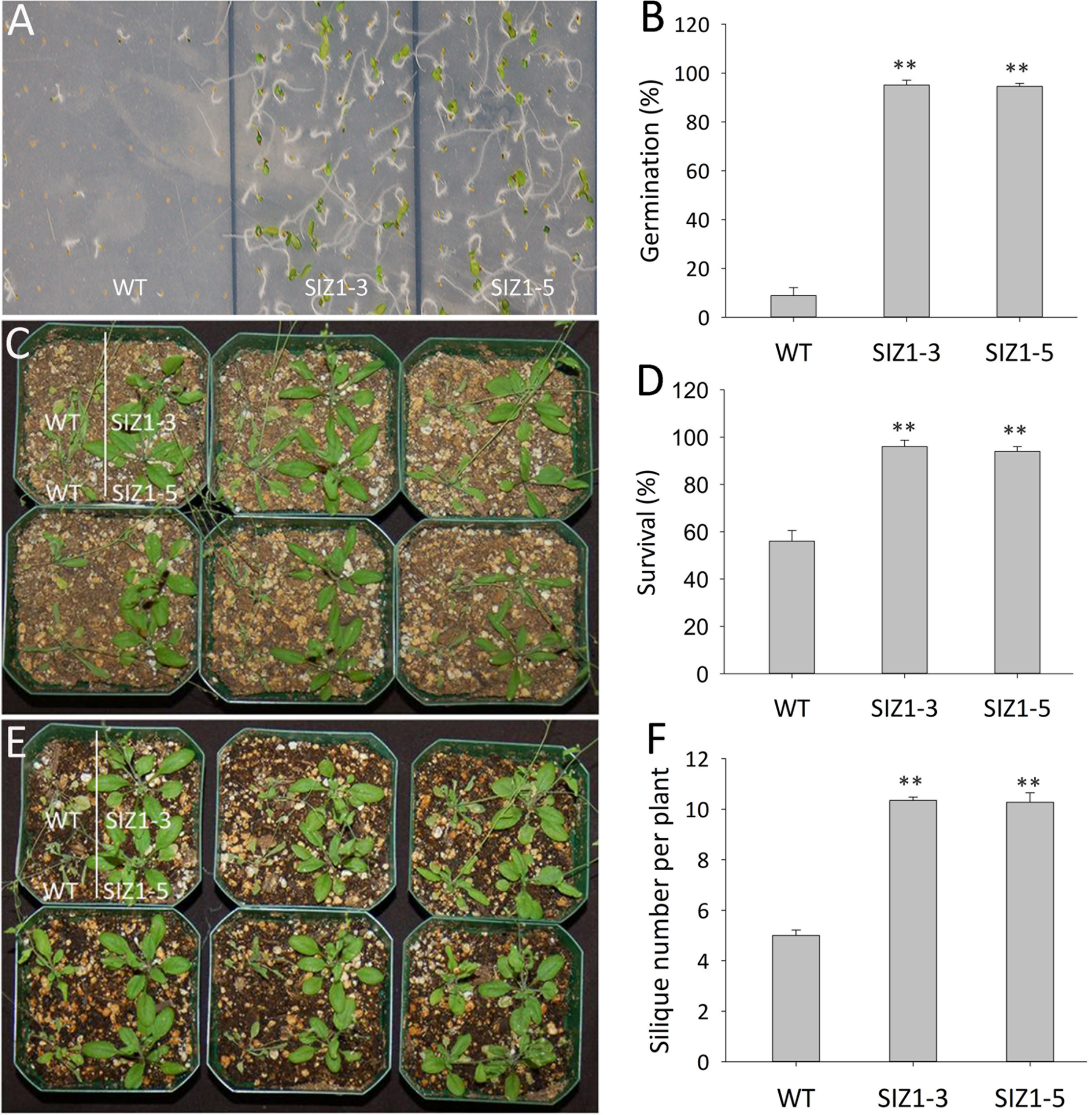
Phenotypes of wild-type and two independent *OsSIZ1*-transgenic plants under combined stresses of heat and water deficit. **A.** Phenotypes of wild-type and two independent *OsSIZ1*-transgenic plants on MS media after treatment of combined stresses of heat and water deficit. Plants were grown on MS media supplemented with 300 mM mannitol and exposed to 37 ^o^C for 48 h immediately after plating. **B.** Germination rates of wild-type and *OsSIZ1*-transgenic plants shown in A. **C.** Phenotypes of wild-type and two independent *OsSIZ1*-transgenic plants in soil under treatment of combined stresses of heat and water deficit. Twenty seven days old plants were exposed to combined stresses of heat and water deficit (no watering for one week and 37 ^o^C for 5 h per day) for a week before the picture was taken. **D.** Survival rate of wild-type and two independent *OsSIZ1*-transgenic plants after exposure to combined stresses of heat and drought. Plants after treatment of combined stresses of heat and salt were re-watered, then the survival rate was determined a week later. **E.** Phenotypes of wild-type and two independent *OsSIZ1*-transgenic plants one week after re-watering following the treatment of combined stresses of heat and water deficit. **F.** Silique numbers of wild-type and two independent *OsSIZ1*-transgenic plants after the treatment of combined stresses of heat and drought. ** statistically significant at 1%; WT, wild-type; SIZ1-3 and SIZ1-5, two independent *OsSIZ1*-transgenic plants.

**Fig 9:**
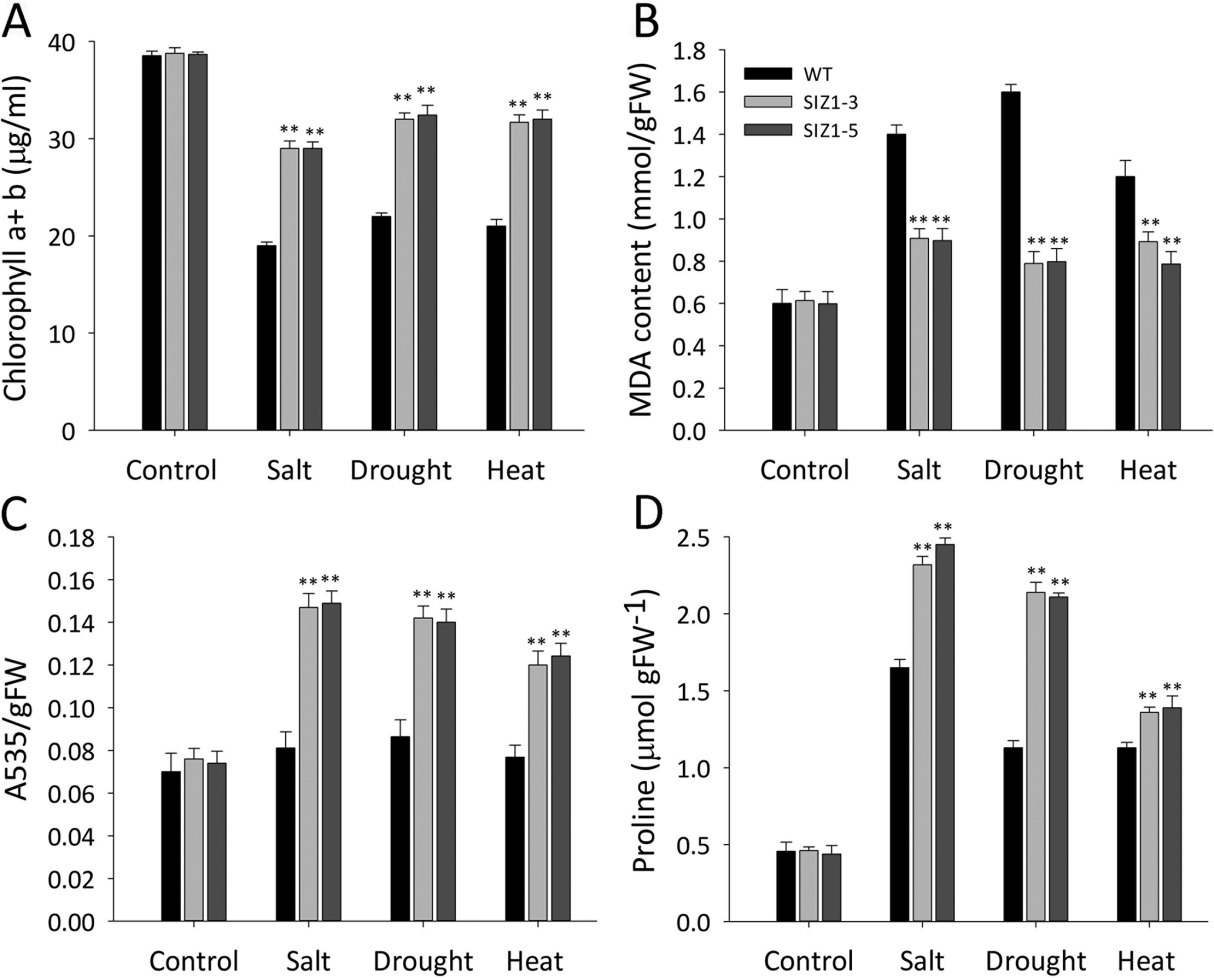
Biochemical responses of wild-type and two independent *OsSIZ1*-transgenic lines to salt, drought, and heat, respectively. **A.** Total chlorophyll content (a + b) in wild-type and *OsSIZ1*-transgenic plants under normal, salt, drought, and heat conditions, respectively. **B.** MDA content in wild-type and *OsSIZ1*-transgenic plants under normal, salt, drought, and heat conditions, respectively. **C.** Anthocyanin content in wild-type and *OsSIZ1*-transgenic plants under normal, salt, drought, and heat conditions, respectively. **D.** Proline content in wild-type and *OsSIZ1*-transgenic plants under normal, salt, drought, and heat conditions, respectively. ** statistically significant at 1%; WT, wild-type; SIZ1-3 and SIZ1-5, two independent *OsSIZ1*-transgenic plants.

**Fig 10:**
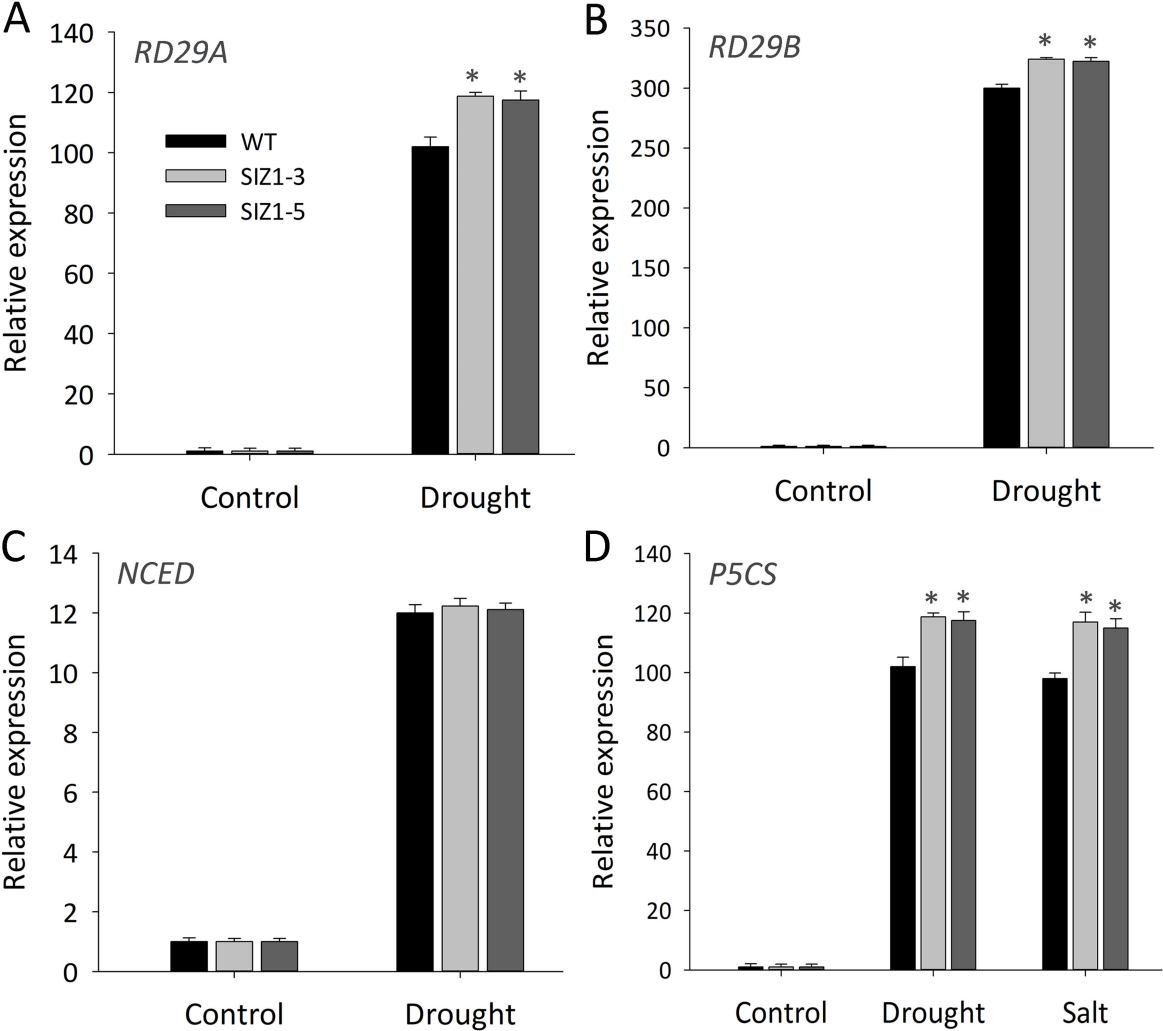
Quantitative real-time PCR analyses of transcripts from several abiotic stress related genes in wild-type and *OsSIZ1*-transgenic plants under drought and salt stresses. **A.** The relative transcript level of *RD29A*. **B.** The relative transcript level of *RD29B*. **C.** The relative transcript level of *NCED*. **D.** The relative transcript level of *P5CS*. * statistically significant at 5%; WT, wild-type; SIZ1-3 and SIZ1-5, two independent *OsSIZ1*-transgenic plants.

### The oligonucleotide primers used in study are:

ACT8_F: TCACCACAACAGCAGAGCGGG

ACT8_R: GGACCTGCCTCATCATACTCGG

SIZ1-1_F: ATGGCGGACCTGGTTTCCag

SIZ1-1_R: ATAGTGACAGTGATTTGGAA

SIZ1-2_F: GCAAAATGGAAATGAACAAA

SIZ1-2_R: CAATAGATACTGATTCTGAGTAG

Actin8_qF: GGAATGGTTAAGGCTGGATTCG

Actin8_qR: CGCATCTTTCTGATTCATCCC

NCED3_F: AAAGCCATCGGTGAGCTTCA

NCED3_R: CAGCTCTGGCGTAGAATAGC

RD29A_F: GCCGACGGGATTTGACG

RD29A_R: GCCGGAAATTTATCCTCTTCTGA

RD29B_F: GGCGGGCAAAGCGAG

RD29B_R: TGCCCGTAAGCAGTAACAGATC

P5CS_F: ATGACGGAGATCGATCGTTCACGC

P5CS_R: CTAAATTCCATTCTCAACAGCCTCTG

## Results

### Creation and molecular characterization of OsSIZ1-overexpressing plants

Wild-type Arabidopsis (Col-0) plants were transformed with Agrobacterium using the floral dip method [43]. A total of 20 independent T_0_ transgenic plants were obtained. Thereafter, T_1_ seeds were screened on MS plates supplemented with 30 μg ml^-1^ BASTA. T_2_ to T_4_ homozygous plants were obtained for transgenic lines by selecting the transgenic plants on MS media supplemented with BASTA. RNA blot analysis was carried out to confirm the lines expressing the *OsSIZ1* gene. One such RNA blot is shown in Fig 1A. Two high expression lines, SIZ1-3 and SIZ1-5, were chosen for further analyses. DNA blot analysis was performed using an *OsSIZ1* cDNA fragment as probe to determine the copy number of transgene. It appears that the SIZ1-5 line contains just one copy of the *OsSIZ*1 transgene (Fig 1B), whereas the SIZ1-3 line displayed three bands in the DNA blot analysis (Fig 1B), indicating the presence of 3 copies of the *OsSIZ1* transgene. However, on MS media supplemented with BASTA, this line gave a 3:1 segregation ratio of resistant plants vs. sensitive plants at T_1_ generation, therefore, it might be a single T-DNA insertion event, but the T-DNA went through a tandem duplication twice before or after insertion.

### Overexpression of OsSIZ1 in Arabidopsis makes plants more tolerant to high temperatures at seedling and adult stages

Higher temperatures can negatively affect all stages of plant life including germination, development, and reproduction [55]. We studied the response of *OsSIZ1*-transgenic plants when grown under higher temperatures. Under normal temperature conditions of around 23 ^o^C, we did not observe significant differences between control and *OsSIZ1*-transgenic plants in hypocotyl length at seedling stage (Fig 2A & 2C). In contrast, the hypocotyl lengths of seedlings exposed to heat stress (45 ^o^C for 5 h) were 57% and 54% longer in transgenic lines SIZ1-3 and SIZ1-5, respectively, when compared to WT plants (Fig 2B & 2C). In addition, the heat stress tolerance in photosynthetically active seedlings was evaluated as a function of seedling survival after imposition of heat stress. Seedlings that remain green and actively growing after heat shock (45 ^o^C for 20 min) were considered to be viable (Fig 2E). It was observed that only 13% of WT seedlings could survive after heat shock, whereas transgenic lines SIZ1-3 and SIZ1-5 displayed 32% and 34% survival rates, respectively (Fig 2F). No significant differences were observed between WT and *OsSIZ1*-transgenic plants when grown under normal temperatures (Fig 2D & 2F). Long term effect of heat stress was also evaluated by exposing soil grown plants under heat stress conditions. Plants were grown under normal condition for 4 weeks and no differences were observed between WT and *OsSIZ1*-transgenic plants (data not shown). Then these plants were transferred to a growth chamber that was set for heat stress treatment (37 ^o^C for 5 h per day), we found that one month after heat stress started, most flowers in WT and *OsSIZ1*-transgenic plants were sterile and only a few siliques could be generated. Despite this, *OsSIZ1*-transgenic lines generated 40–43% higher seed yield than WT plants under that condition (Fig 2H & 2I). No significant differences in seed yield were observed between WT and *OsSIZ1*-transgenic plants when grown under normal temperature conditions (22 ^o^C) (Fig 2G & 2I).

### Overexpression of OsSIZ1 significantly improves salt tolerance in Arabidopsis

We analyzed the salt tolerance of WT and *OsSIZ1*-transgenic plants using short term assays on MS plates and long term assays with soil grown plants. We did not observe significant differences in root lengths between WT and *OsSIZ1*-transgenic plants when plants were grown on MS media without NaCl (Fig 3A). However, with 125 mM NaCl in media, *OsSIZ1*-transgenic plants performed significantly better than WT plants by having 52–53% longer root length (Fig 3B & 3D). With 150 mM NaCl media, more than 90% of WT seedlings became chlorotic after one week of salt treatment, generating ~70% less fresh weight than *OsSIZ1*-transgenic plants (Fig 3C & 3E). For soil grown plants, before starting the salt stress treatments, no differences were observed between the WT and *OsSIZ1*-transgenic plants (S1A Fig). However, after incremental application of salt solution (50 mM NaCl twice in 6 days, 100 mM NaCl twice in the next 6 days, and 150 mM NaCl three times in the next 9 days), WT plants became withered due to salt stress while *OsSIZ1-*transgenic plants were much healthier. *OsSIZ1*-transgenic plants generated ~79% greater seed yield than WT plants (Fig 3G & 3H). No significant differences could be detected in seed yield for WT and *OsSIZ1*-transgenic plants grown under normal conditions (Fig 3F & 3H).

### OsSIZ1-transgenic plants exclude Na^+^ ions from cytoplasm better than WT plants and accumulate higher K^+^ ions

To investigate the effects of *OsSIZ1*-overexpression on ion contents in transgenic plants, sodium (Na^+^) and potassium (K^+^) contents were analyzed in roots and shoots of WT and *OsSIZ1*-transgenic plants grown under normal and salt stress conditions. It was found that WT plants accumulated ~40% higher Na^+^ content in shoots (Fig 4A) and ~45% higher Na^+^ content in roots (Fig 4B) as compared to *OsSIZ1*-transgenic plants. It appears that *OsSIZ1*-transgenic plants have higher ability to exclude Na^+^ ions from plant cells than WT plants. In addition, *OsSIZ1*-transgenic plants accumulated ~20% and ~33% higher K^+^ ion content in shoots and roots, respectively, than WT plants (Fig 4C & 4D), indicating that K^+^ ion accumulation might be one of the reasons for the improved performance of *OsSIZ1*-transgenic plants in saline soils. No differences were observed for Na^+^ and K^+^ contents in roots and shoots between WT and *OsSIZ1*-transgenic plants grown under normal conditions (Fig 4A to 4D).

### Overexpression of OsSIZ1 in Arabidopsis significantly improves germination and seed yield under water deficit conditions

To study the effect of water deficit stress on seed germination, seeds of WT plants and two *OsSIZ1*-transgenic lines were plated on MS media containing polyethylene glycol (PEG-8000). PEG is known to induce low water potential in growth media [50], thereby creating a water deficit stress condition. Because PEG prevents polymerization of agar, it was added as an overlay solution to agar media (sterilized liquid MS media with PEG added after autoclaving). Forty percent PEG-8000 (0.5M) was added to the MS media, creating a water potential of -0.7 MPa. We did not observe differences in germination between WT and *OsSIZ1*-transgenic plants when grown on MS media without PEG (Fig 5A & 5C), but germination was significantly affected with the addition of PEG in the media. The *OsSIZ1-*transgenic lines showed an average of 64% higher germination rate as compared to that of WT plants (Fig 5B & 5C).

Long term effect of water deficit stress was investigated with soil grown plants. Three and a half weeks after germination, plants were fully watered and no differences could be distinguished between *OsSIZ1*-transgenic and WT plants (S1B Fig). However, once watering was stopped for two weeks, significant differences were seen between *OsSIZ1*-transgenic and WT plants (Fig 5D). Thereafter, plants were allowed to recover for 24 h after re-watering, almost 50% of WT plants could not recover, yet ~ 95% of *OsSIZ1*-transgenic plants recovered from drought stress (Fig 5E). Furthermore, *OsSIZ1*-transgenic plants generated 64% higher seed yield than WT plants (Fig 5F). In comparison, no differences were observed between WT and *OsSIZ1*-transgenic plants when grown under full irrigation conditions (S2 Fig).

### OsSIZ1-transgenic plants outperformed wild-type plants under combined stresses of heat and salt

In nature, multiple stresses can occur simultaneously [56]. Moreover, salinity stress gets intensified under high temperature conditions due to increased evapo-transpiration [57], causing a greater loss in crop yields. To test how *OsSIZ1*-transgenic plants would perform under multiple stress conditions, seeds of WT and *OsSIZ1*-transgenic plants were plated on MS media supplemented with 100 mM NaCl and plates were kept in growth chamber adjusted to 30 ^o^C as opposed to an optimal temperature of 22 ^o^C. It was observed that after 2 weeks of growth, the root length of WT plants were significantly reduced as compared to that of *OsSIZ1*-transgenic plants (Fig 6B & 6C). In contrast, no significant differences in root length could be seen between WT and *OsSIZ1*-transgenic seedlings grown under normal conditions (i.e. MS plates without NaCl and kept at 22 ^o^C, Fig 6A). For plants grown in soil for 4.5 weeks under normal conditions, no phenotypic differences were observed (Fig 7A). Then these plants were transferred to a growth chamber that was set at 37 ^o^C for 5 h per day (12:00 noon to 5:00 pm), and salt was applied incrementally from 50 mM NaCl (three times in 6 days) to 100 mM NaCl (three times in the next 6 days). We then found that the growth of WT plants was severely inhibited, many were dead, and those survived produced fewer siliques (Fig 7B & 7C). In contrast, *OsSIZ*1-transgenic plants were much larger in sizes, 100% survived, and produced far more siliques (Fig 7C & 7D).

### OsSIZ1-transgenic plants demonstrated enhanced performance than wild-type plants under combined stresses of heat and water deficit

Heat and drought stresses often occur at the same time, therefore they are the most common combination of abiotic stresses in nature, which can cause detrimental effects on crop yields [56]. To study the effect of combined heat and drought/osmotic stresses on plants, seeds of WT and two *OsSIZ1-*transgenic lines were directly plated on MS media supplemented with 300 mM mannitol and soon after plating, plates were kept in an incubator that was set at 37 ^o^C for 48 h, then these plates were returned to normal temperature condition (22 ^o^C). We observed that even two weeks after returning to normal temperature, only 10–15% of WT seeds germinated, however almost ~100% of *OsSIZ1*-transgenic plants germinated (Fig 8A & 8B). No differences in germination could be seen between WT and *OsSIZ1*-transgenic plants grown under normal conditions (MS media) and under mannitol caused osmotic stress conditions (MS media with 300 mM mannitol) (data not shown). To study the long-term effect of combined stresses of heat and drought, WT and Os*SIZ1*-transgenic plants were grown in soil under normal conditions for 4.5 weeks (picture not shown), then plants were transferred to a growth chamber that was set at 37 ^o^C for 5 h every day and watering was stopped for a week. We observed that almost half of WT plants died, and the remaining ones were severely withered, yet 100% of *OsSIZ1*-transgenic plants survived under this condition (Fig 8C & 8D). Also, most WT plants that survived could not recover after re-watering (Fig 8E), and the ones that did recover produced ~70% less yield than *OsSIZ1*-transgenic plants (Fig 8F). No significant differences were observed between WT and *OsSIZ1*-transgenic plants grown in soil under normal conditions (data not shown).

### OsSIZ1-transgenic plants maintained higher chlorophyll content than wild-type plants under abiotic stress conditions

One of the major consequences of abiotic stresses in plants is chlorophyll degradation [58]. Under salt, drought, and heat stress conditions, *OsSIZ1-*transgenic plants looked healthier and greener than WT plants. One primary reason for the better health of *OsSIZ*1-transgenic plants under these stress conditions could be due to lower degradation of chlorophyll. Indeed, we found that *OsSIZ1*-transgenic plants maintained 34%, 31%, and 32% higher chlorophyll content under salt, drought, and heat stress conditions, respectively (Fig 9A).

### OsSIZ1-transgenic plants are more efficient in scavenging reactive oxygen species and have higher proline content

Abiotic stresses usually lead to production of reactive oxygen species (ROS) that in turn cause oxidative damages in plants [56]. Increased peroxidation and degradation of lipids were reported in plants growing under environmental stresses, and malondialdehyde (MDA) is one of the final products of peroxidation of unsaturated fatty acids in phospholipids [59]. We observed that WT plants produced 35%, 50%, and 30% higher MDA content under salt, drought, and heat stress conditions, respectively (Fig 9B). To alleviate the damages caused by ROS, plants have evolved several mechanisms to scavenge ROS. One scavenger molecule of ROS is anthocyanin, a flavonoid, which is an important molecule responsible for detoxification of ROS. We observed that *OsSIZ1*-transgenic plants accumulated 42%, 38% and 37% more anthocyanin as compared to WT plants under salt, drought, and heat stress conditions, respectively (Fig 9C). Increased production of anthocyanin, a ROS scavenger, might be one of the reasons for decreased MDA content in *OsSIZ1*-transgenic plants. Furthermore, *OsSIZ1*-transgenic plants were shown to have significantly higher proline content than wild-type plants under salt, drought, and heat stress conditions, respectively (Fig 9D). Because proline is an amino acid that can protect plant cells under various abiotic stress conditions [60, 61], therefore *OsSIZ1*-transgenic plants have a higher capacity to deal with ROS under abiotic stress conditions.

### OsSIZ1-transgenic plants do not respond to osmotic stress alone

To test if *OsSIZ1* overexpression is involved in enhancing osmotic stress tolerance in Arabidopsis, three days old seedlings of wild-type and *OsSIZ1*-transgenic lines were transferred to MS plates supplemented with 250 mM mannitol, a non-metabolizable sugar that could cause non-ionic osmotic stress in plants. We did not observe significant differences between wild-type and *OsSIZ1*-transgenic plants in root length on MS media with or without mannitol (S3 Fig). However, root lengths were significantly reduced in the presence of mannitol in all genotypes as compared to under normal growth conditions (S3 Fig). *OsSIZ1*-transgenic plants do not respond to mannitol caused osmotic stress alone in root growth at seedling stage (S3B Fig). However, when osmotic stress imposed by mannitol was combined with heat stress, germination was significantly inhibited in WT plants as compared to *OsSIZ1*-transgenic plants (Fig 8A).

### Enhanced expression of stress related genes in OsSIZ1-transgenic plants under abiotic stress conditions

Both drought stress and salt stress impose an osmotic stress that can lead to loss of turgor in plant cells [62]. Plants respond to these stresses by activating expression of a group of stress related genes. We found that the transcript levels of *RD29A* and *RD29B* were significantly higher in *OsSIZ1-*transgenic plants than wild-type plants (Fig 10A & 10B). However, we did not see significant differences in the transcript level of *NCED3*, a critical gene in ABA biosynthetic pathway [63, 64], between wild-type and *OsSIZ1*-transgenic plants under drought stress conditions (Fig 10C). The expression of *P5CS* that encodes the pyrroline 5-carboxylate synthase (a critical enzyme in the biosynthesis of proline) was reported to be increased by various abiotic stresses [65], and indeed our real time PCR analysis indicated that the *P5CS* transcript was higher in *OsSIZ1*-transgenic plants under salt and drought stress conditions (Fig 10D).

### Seed viability is compromised after exposure to high temperature and salinity stresses

We analyzed how seeds harvested from plants grown under stress conditions would germinate on MS media after stratification. We found that seeds harvested from *OsSIZ1*-transgenic plants exposed to salinity and heat stress displayed significantly better germination than seeds from WT plants (S4 Fig), yet no significant differences in seed germination could be seen between wild-type and *OsSIZ1*-transgenic plants exposed to drought stress alone (S4 Fig).

## Discussion

Drought, heat and salt stresses are the three major abiotic stresses adversely affecting crop yields worldwide. In natural conditions, these stresses come alone or in various combinations at different stages of plant development. Considering the devastating effects caused by the multiple stresses, there is an urgent need to develop crops that can tolerate multiple stresses with minimal yield loss. Here we report that transgenic Arabidopsis plants expressing *OsSIZ1* display improved tolerance to salt, heat and drought separately and in combinations as compared to wild-type plants. Under heat stress conditions, transgenic plants outperformed wild-type plants by displaying better hypocotyl length, seedling survival and seed yield (Fig 2). Under salt stress conditions, *OsSIZ1*-transgenic plants demonstrated better root growth and higher seed yield than wild-type plants (Fig 3), and they are able to exclude Na^+^ more efficiently than wild-type plants (Fig 4). Under drought stress, transgenic plants outperformed wild-type plants by exhibiting better germination rates and higher seed yield (Fig 5). Also, *OsSIZ1*-transgenic plants performed significantly better than wild-type plants under combined stress conditions such as heat plus salt (Figs 6 & 7) and heat plus drought (Fig 8). Although it was known that overexpression of *OsSIZ1* could increase drought and heat tolerance in transgenic plants [36, 37], discovering that overexpression of *OsSIZ1* could also increase salt tolerance was a surprise to us. Transgenic plants expressing *OsSIZ1* gene were found to tolerate abiotic stresses much better than those of wild type plants. At the same time, they were able to maintain the seed yield or showed less penalty than wild type plants in terms of seed yield. It is really a good candidate to improve production in cereals where seed is economically important part of plants. Our study with overexpression of *OsSIZ1* in Arabidopsis indicate that there does not appear a penalty for transgenic plants under normal growth conditions, yet under salt, drought, and heat stress conditions, overexpression of *OsSIZ1* provides an indisputable edge for transgenic plants, an indication that the *OsSIZ*1 gene has the great potential to be used for crop’s improvement.

One of major consequences of plant response to abiotic stresses is the production of ROS. Accumulation of ROS in plant cells can lead to oxidation of various cellular components such as proteins and lipids, leading to enzyme inactivation or inhibition, resulting in degradation of chlorophyll and eventually cell death [66]. *OsSIZ1*-transgenic plants are able to handle ROS more effectively as they can maintain higher chlorophyll content under salt, drought, and heat conditions (Fig 9A). One consequence of plant response to abiotic stresses is the production of MDA, a product of lipid peroxidation by ROS [67]. In this study, we found that although MDA content increased under salt, drought, and heat stress conditions, *OsSIZ1*-transgenic plants produced much less MDA as compared to that of wild-type plants (Fig 9B), again supporting that transgenic plants can scavenge ROS better than those of wild-type plants. One plausible reason for *OsSIZ1*-transgenic plants to scavenge ROS more efficiently is that they have higher capacity to remove ROS. This hypothesis is supported by the fact that *OsSIZ1*-transgenic plants produced more anthocyanin pigment, an effective ROS scavenger under stress conditions (Fig 9C). Our study with *OsSIZ1*-tranegnic cotton showed that transcript levels of several antioxidant genes such as *APX*, *SOD*, and *GST* were higher in *OsSIZ1*-transgenic cotton plants [37], a result that is consistent with the discovery in this study. Production of osmoprotectants is one of the strategies adopted by plants to cope up with the damaging effects of water deficit stress. Both drought and salt stress impose conditions of dehydration stress, as under drought stress, no water is available to plants, and under salt stress, plants cannot take up water from soil due to decreased water potential in soil solution. Osmoprotectants such as proline are known to reduce the cellular water potential, thereby preserving water in the cell and maintaining turgor pressure in plant cells [68]. Under salt, drought, and heat stress conditions, *OsSIZ1*-transgenic plants are able to maintain a higher level of proline (Fig 9D), another reason why *OsSIZ1*-transgenic plants can perform better than wild-type plants under salt, drought and heat stress conditions.

It is not completely clear why overexpression of *OsSIZ1* could confer multi-stress tolerance in transgenic plants. Because SIZ1 protein is located in the nucleus of plant cells [69–72], it is likely that one of the major functions of SIZ1 is to activate transcriptional factors that in turn activate expression of a battery of stress related genes. It was shown that AtSIZ1 could regulate the transcriptional factor ABI5 and ICE1 [24, 31] a clear indication that SIZ1 serves as a master regulator that controls expression of many genes. We analyzed transcripts of a few stress related genes and we found that their transcripts were indeed higher in *OsSIZ1*-transgenic plants than those in wild-type plants. For example, the transcripts of *RD29A* and *RD29B* were enhanced in *OsSIZ1*-transgenic plants (Fig 10A and 10B). The transcript of *P5CS*, a gene encoding the rate limiting enzyme in the biosynthesis of proline, was also higher in *OsSIZ1*-transgenic plants than that in wild-type plants (Fig 10D), which explains why the proline content was higher in *OsSIZ1*-transgenic plants (Fig 9D).

Because the Arabidopsis *siz1* mutant displays multi-facet deficiencies in plant growth and development, plant response to abiotic stresses, and plant cellular metabolism [4, 32] the increased multi-stress tolerance by overexpression of *OsSIZ1* transgenic plants could involve several potential mechanisms. For example, *OsSIZ1* transgenic plants could exclude more Na^+^ from the cytoplasm and at the same time accumulate higher amounts of K^+^ ions in both roots and shoots (Fig 4), suggesting that *OsSIZ1*-transgenic plants might use both low- and high-affinity systems for K^+^ uptake. Sodium ions have a more damaging effect as once Na^+^ gets into the cytoplasm, it inhibits the activities of many enzymes [73]. Therefore, we speculate that SIZ1 can activate either K^+^ transporters such as HKT1 or Na^+^/H^+^ antiporters on the plasma membrane such as SOS1. However, further studies are needed to confirm the potential action of SIZ1 (or OsSIZ1) on ion transporters and antiporters. Although we could not provide a mechanistic understanding of *OsSIZ1*-mediated multi-stress tolerance in transgenic plants at this time, the benefits of *OsSIZ1* overexpression in transgenic plants are obvious.

## Supporting information

S1 Fig Phenotypes of wild-type and two independent *OsSIZ1*-transgenic plants before stress treatments in soil. **A.** Three and a half weeks old Arabidopsis plants before the salt stress treatment. **B.** Three and a half weeks old Arabidopsis plants before the drought stress treatment. WT, wild-type; SIZ1-3 and SIZ1-5, two independent *OsSIZ1*-transgenic plants.
(TIF)

S2 Fig Phenotypes of wild-type and *OsSIZ1*-transgenic plants grown in soil under normal conditions. Plants were grown for 45 days under normal condition (no stress and irrigation with regular water). WT, wild-type; SIZ1-3 and SIZ1-5, two independent *OsSIZ1*-transgenic plants.
(TIF)

S3 Fig Phenotypes of wild-type and *OsSIZ1*-transgenic plants in the presence of mannitol. **A.** Phenotypes of wild-type and *OsSIZ1*-transgenic plants in the absence of mannitol. Three days old seedlings were transferred to MS to grow vertically for 10 days. **B.** Phenotypes of wild-type and *OsSIZ1*-transgenic plants in the presence of mannitol. Three days old seedlings were transferred to MS plates that contain 250 mM mannitol to grow vertically for 10 days. **C.** Analysis of root lengths of wild-type and *OsSIZ1*-transgenic plants in the presence or absence of mannitol. Three days old seedlings were transferred to MS plates to grow for 7 days in the absence of mannitol (black bars) or presence of 250 mM of mannitol (gray bars). WT, wild-type; SIZ1-3 and SIZ1-5, two independent *OsSIZ1*-transgenic plants.
(TIF)

S4 Fig Germination rates of seeds harvested from wild-type and *OsSIZ1*-transgenic plants grown under normal (no stress), salt stress, drought stress, and heat stress conditions. WT, wild-type; SIZ1-3 and SIZ1-5, two independent *OsSIZ1*-transgenic plants.
(TIF)
