# Supplementary figures and images for: Overexpression of the rice gene OsSIZ1 in Arabidopsis improves drought-, heat-, and salt-tolerance simultaneously
Source: PLoS One. 2018 Aug 9;13(8):e0201716. doi: 10.1371/journal.pone.0201716 (PMC6084956; doi:10.1371/journal.pone.0201716)

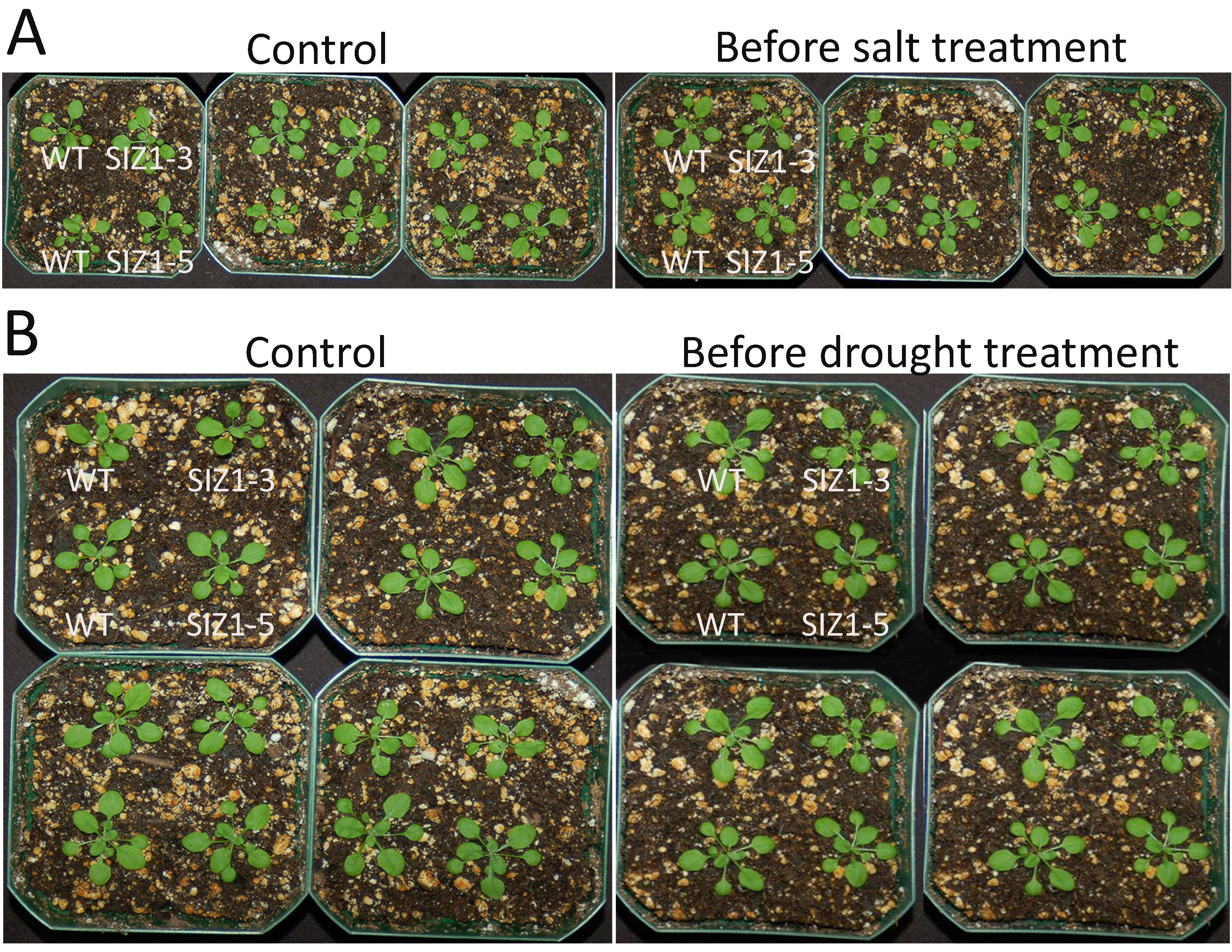

Supplement: S1 Fig — A. Three and a half weeks old Arabidopsis plants before the salt stress treatment. B. Three and a half weeks old Arabidopsis plants before the drought stress treatment. WT, wild-type; SIZ1-3 and SIZ1-5, two independent OsSIZ1-transgenic plants. (TIF) [file pone.0201716.s001.tif]

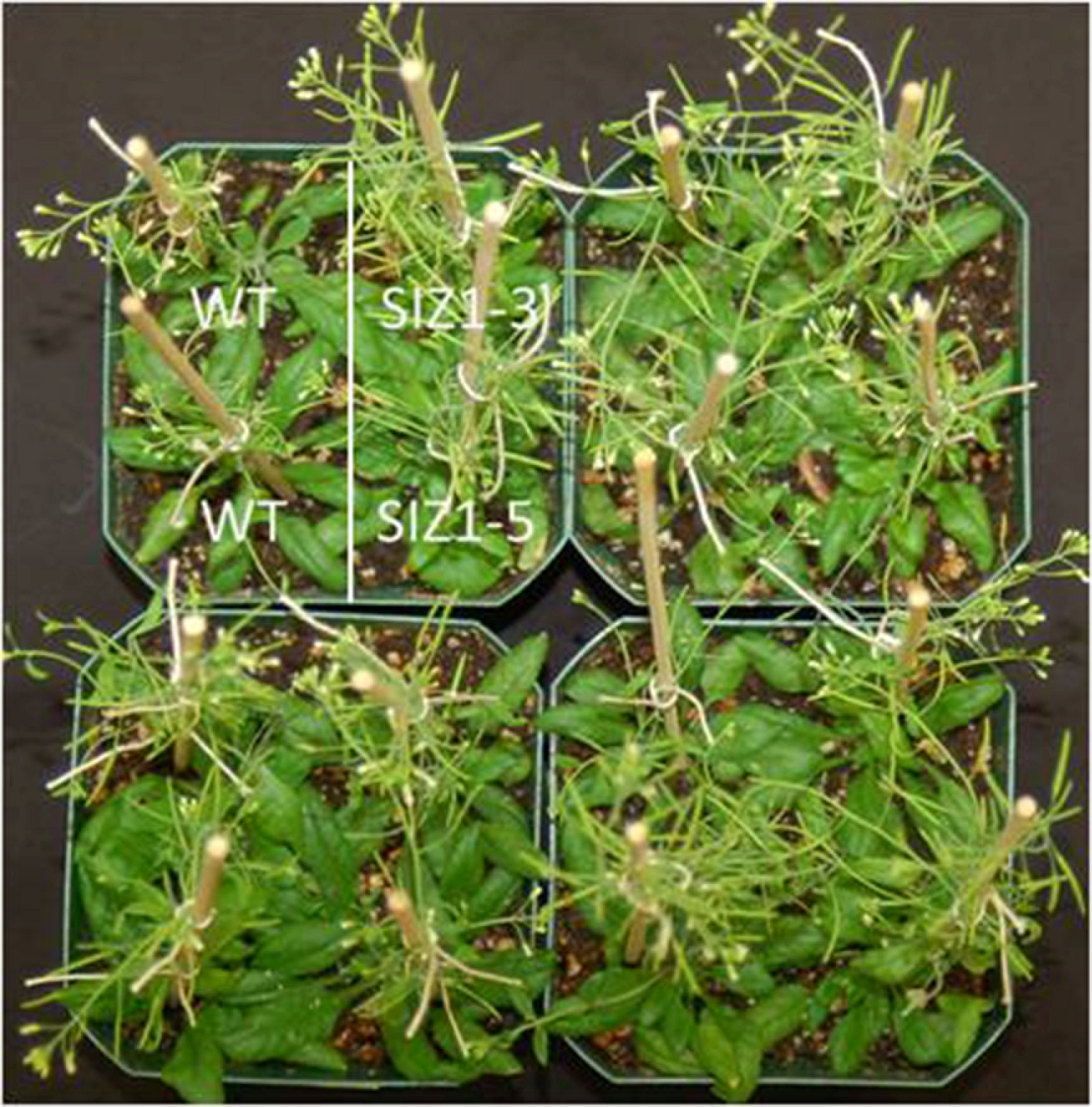

Supplement: S2 Fig — Plants were grown for 45 days under normal condition (no stress and irrigation with regular water). WT, wild-type; SIZ1-3 and SIZ1-5, two independent OsSIZ1-transgenic plants. (TIF) [file pone.0201716.s002.tif]

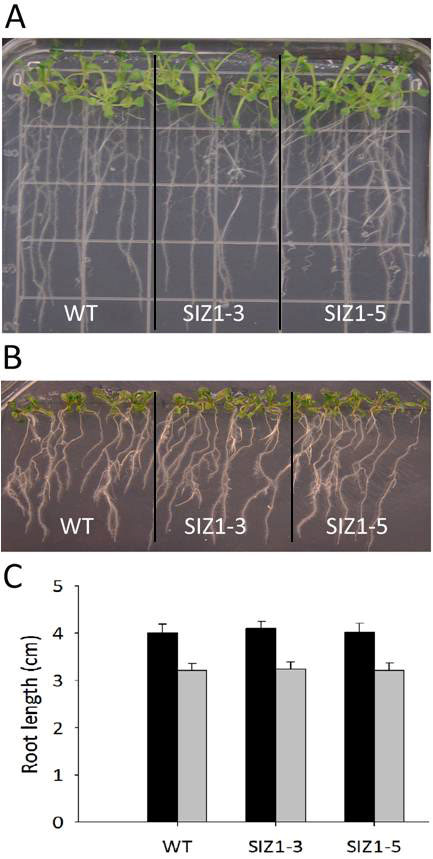

Supplement: S3 Fig — A. Phenotypes of wild-type and OsSIZ1-transgenic plants in the absence of mannitol. Three days old seedlings were transferred to MS to grow vertically for 10 days. B. Phenotypes of wild-type and OsSIZ1-transgenic plants in the presence of mannitol. Three days old seedlings were transferred to MS plates that contain 250 mM mannitol to grow vertically for 10 days. C. Analysis of root lengths of wild-type and OsSIZ1-transgenic plants in the presence or absence of mannitol. Three days old seedlings were transferred to MS plates to grow for 7 days in the absence of mannitol (black bars) or presence of 250 mM of mannitol (gray bars). WT, wild-type; SIZ1-3 and SIZ1-5, two independent OsSIZ1-transgenic plants. (TIF) [file pone.0201716.s003.tif]

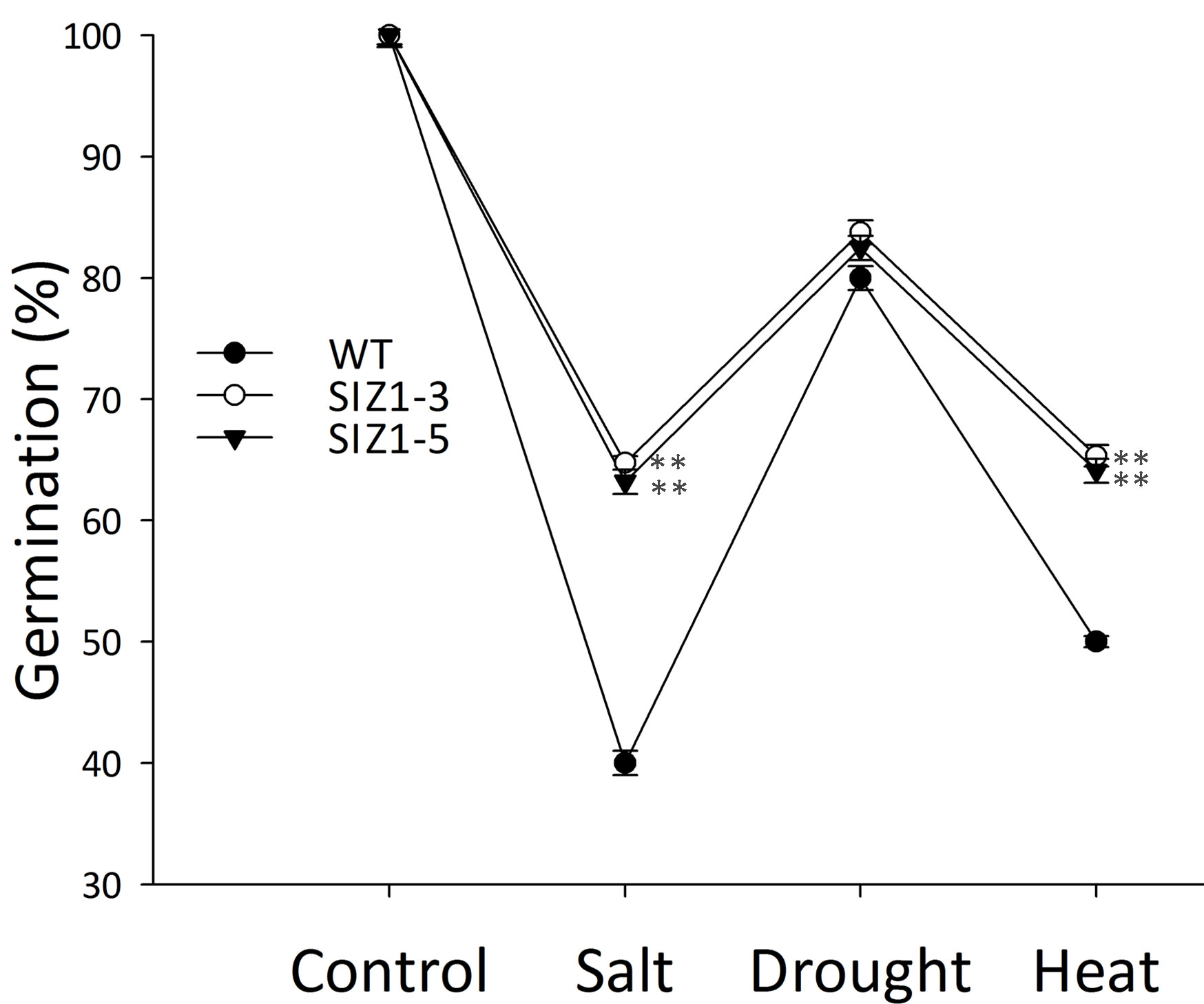

Supplement: S4 Fig — WT, wild-type; SIZ1-3 and SIZ1-5, two independent OsSIZ1-transgenic plants. (TIF) [file pone.0201716.s004.tif]
